# Revealing phenotype-associated functional differences by genome-wide scan of ancient haplotype blocks

**DOI:** 10.1371/journal.pone.0176530

**Published:** 2017-04-26

**Authors:** Ritsuko Onuki, Rui Yamaguchi, Tetsuo Shibuya, Minoru Kanehisa, Susumu Goto

**Affiliations:** 1 Bioinformatics Team, Advanced Analysis Center, National Agriculture and Food Research Organization (NARO), 2-1-2 Kannondai, Tsukuba, Ibaraki, Japan; 2 Human Genome Center, Institute of Medical Science, University of Tokyo, 4-6-1 Shirokanedai, Minato-ku, Tokyo, Japan; 3 Bioinformatics Center, Institute for Chemical Research, Kyoto University, Gokasho, Uji, Kyoto, Japan; Universidade Nova de Lisboa Instituto de Higiene e Medicina Tropical, PORTUGAL

## Abstract

Genome-wide scans for positive selection have become important for genomic medicine, and many studies aim to find genomic regions affected by positive selection that are associated with risk allele variations among populations. Most such studies are designed to detect recent positive selection. However, we hypothesize that ancient positive selection is also important for adaptation to pathogens, and has affected current immune-mediated common diseases. Based on this hypothesis, we developed a novel linkage disequilibrium-based pipeline, which aims to detect regions associated with ancient positive selection across populations from single nucleotide polymorphism (SNP) data. By applying this pipeline to the genotypes in the International HapMap project database, we show that genes in the detected regions are enriched in pathways related to the immune system and infectious diseases. The detected regions also contain SNPs reported to be associated with cancers and metabolic diseases, obesity-related traits, type 2 diabetes, and allergic sensitization. These SNPs were further mapped to biological pathways to determine the associations between phenotypes and molecular functions. Assessments of candidate regions to identify functions associated with variations in incidence rates of these diseases are needed in the future.

## Introduction

Genome-wide scans of positive selection are a recent advance in genomic medicine, and have become an important way to infer risk allele variations across populations and elucidate genetic mechanisms of human evolutionary adaptation to local environments, dietary patterns, and infectious diseases [1]. Because detection of positive selection will help improve population-specific disease prevention strategies and treatments, many previous studies revealed that risk alleles for common complex diseases show substantial variation across human populations and contribute to disease risk variation among populations [2–8]. For example, risk alleles for type 2 diabetes (T2D) show high frequencies in African populations and low frequencies in Asian populations [8]. The patterns of risk allele frequencies are shown to be consistent with the disparity in T2D risk across populations of different ancestries, which is thought to be due to adaptations to different agricultural developments across continents. If we know populations have a higher T2D risk (e.g., African ancestry), we can take population-specific preventive actions for T2D based on the genetic background of individuals. Another well-known example is cytochrome P450 (CYP) genes [9]. The allele of an SNP in CYP3A5, a member of the CYP3A subfamily, shows large frequency differences between African Americans and non-Africans [9–11]; and the region that contains this gene also shows a high degree of linkage disequilibrium (LD) that was affected by positive selection in Europeans [9, 12]. Because this allele is involved in CYP3A5 expression and metabolism of clinically important drugs (e.g., the immunosuppressant tacrolimus [13] and the HIV protease inhibitor saquinavir [14]), differences in genetic background may be associated with differential drug responses among populations [9–11]. Other common complex diseases with risk allele frequencies that differ across human populations include cancers (e.g., breast cancer and prostate cancer), cardiovascular diseases, metabolic diseases (e.g., hypertension), neurodegenerative diseases (e.g., Alzheimer’s disease), and systemic autoimmune diseases (e.g., systemic lupus erythematosus and rheumatoid arthritis) [3, 15].

Whereas most studies have focused on recent positive selection, ancient human adaptation to pathogens is known to have affected the immune system and is also associated with risk allele frequency variation for common diseases, such as autoimmune and metabolic disorders among populations [16]. It was reported that ancient local adaptation to pathogens affected celiac disease, type I diabetes, and multiple sclerosis susceptibility loci [17]. It was also reported that ancient selection in response to a sleeping sickness pathogen in Africa contributed to the high rate of renal disease in African Americans [18]. Another example is adaptation to malaria pathogens, *Plasmodium* spp., which appeared more than 100,000 years ago (100 kya) in Africa. Most malaria resistance alleles occur in African populations, and the LD segments associated with the alleles are short and highly variable between populations [16]; however, whether variation among populations affects the incidence of recent common diseases has not been well documented [19]. Therefore, in addition to recent positive selection, ancient positive selection is important for detecting immune-mediated common diseases.

Approaches to finding positively selected regions in the human genome are classified into four groups [20]: summary statistics, LD-based statistics [21–26], comparative genomics, and neutrality tests. These approaches are mainly applied to detect recent positive selection. For example, positive selection signals of the lactase persistence allele at the LCT locus were detected by long haplotype tests (i.e., LD-based approaches such as LRH, iHS, and XP-EHH) [27, 28]. XP-EHH [28] also detected positive selection of SLC24A5 that is associated with skin pigment differences among populations. Significant variations in T2D risk alleles across populations have been revealed using iHS and XP-EHH [8, 29, 30]. These methods aim to identify positive selection that occurred after dispersal out of Africa (< 30 kya) [27, 28], and the mean lengths of detected regions are more than 400 kb. Recently, selection events have been detected in the ancestral population of all present-day humans [31–33], and 3P-CLP [34] was developed to detect ancient selection events that occurred before the split of Yoruba and Eurasians but after their split from Neanderthals.

**Fig 1:**
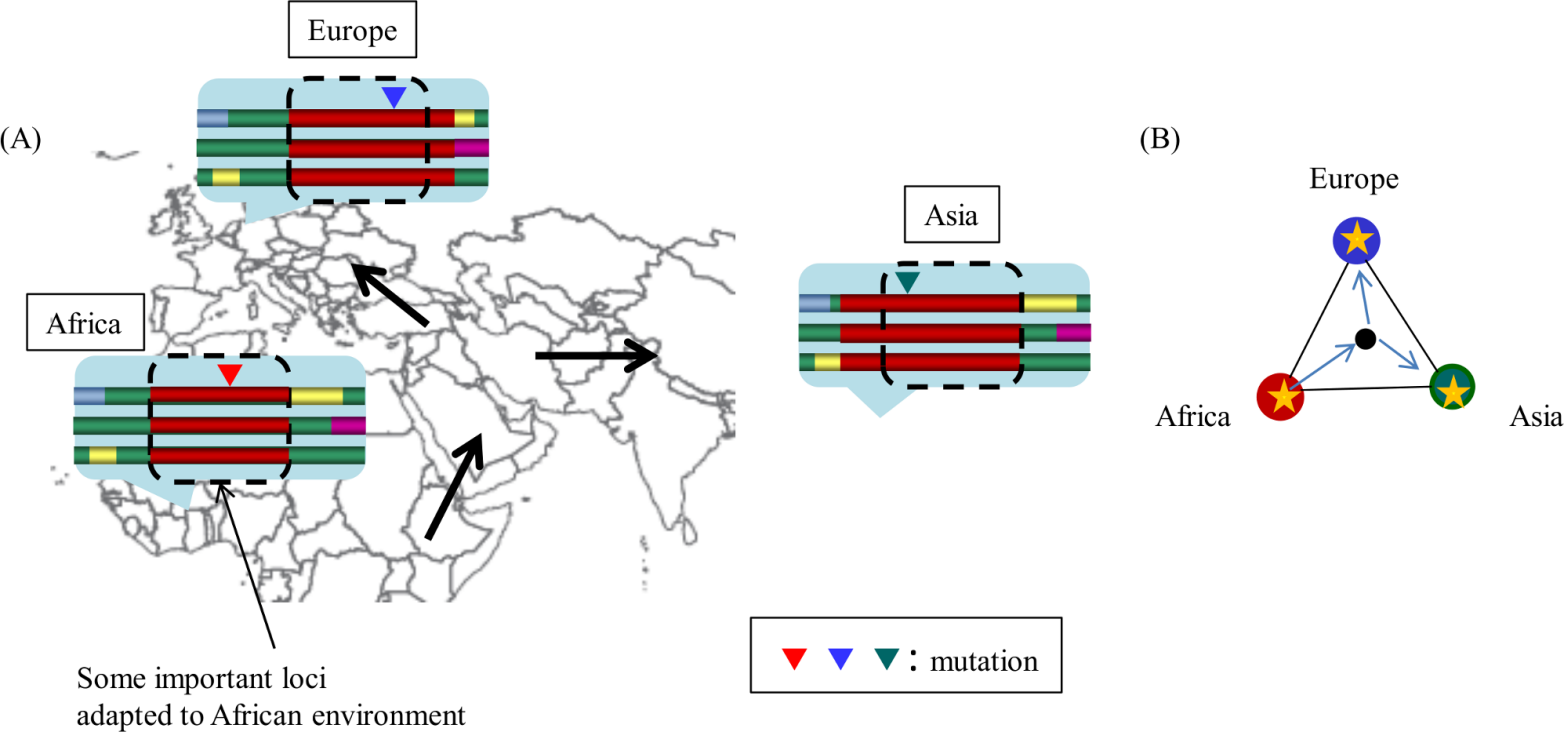
Signatures of ancient haplotype blocks with population-specific positive selection. (A) Some important loci adapted to ancient African environment arose (red triangle) and formed haplotype blocks. The haplotype blocks spread during human migration, and some mutations may have occurred for adaptation to each environment (blue and green triangles). This change is a signature of an ancient haplotype block with population-specific positive selection. (B) A proposed network model to represent the positive selection signature. Each node represents the population in a region. Throughout this paper, red, blue, and green nodes represent populations in Africa, Europe, and Asia, respectively. Arrows represent migration routes. Edges represent relationships between populations. In this work, relationships were evaluated using t-statistic scores that represent degrees of difference between populations. Asterisks represent mutations.

In this study, we develop a pipeline to detect ancient positive selection events. We use the term ‘ancient’ to describe the period before the human migrations out of Africa (~100 kya). We hypothesize that haplotype blocks, i.e., conserved regions, that contain variants that were selected in ancient times have spread with human migration, and some mutations occurred for adaptation to each local environment (Fig 1). This pipeline first identifies ancient haplotype blocks by screening common blocks after extracting those within each population. The pipeline then scans the identified ancient haplotype blocks to check whether they have haplotype frequency variation among populations.

After extracting ancient haplotype blocks with haplotype frequency variation across populations by applying the pipeline to HapMap2 genotype data [35], we annotated the genes in the extracted blocks using the Kyoto Encyclopedia of Genes and Genomes (KEGG) pathway database [36], and identified genes associated with immune system-related functions that are potentially related to common diseases. We also analyzed SNPs in the blocks using the NHGRI GWAS catalog [37] to infer the relationships among SNPs, diseases, and genes whose biological functions are described by functional categories in the KEGG pathway database.

## Materials and methods

### HapMap data for genome-wide scan

**Fig 2:**
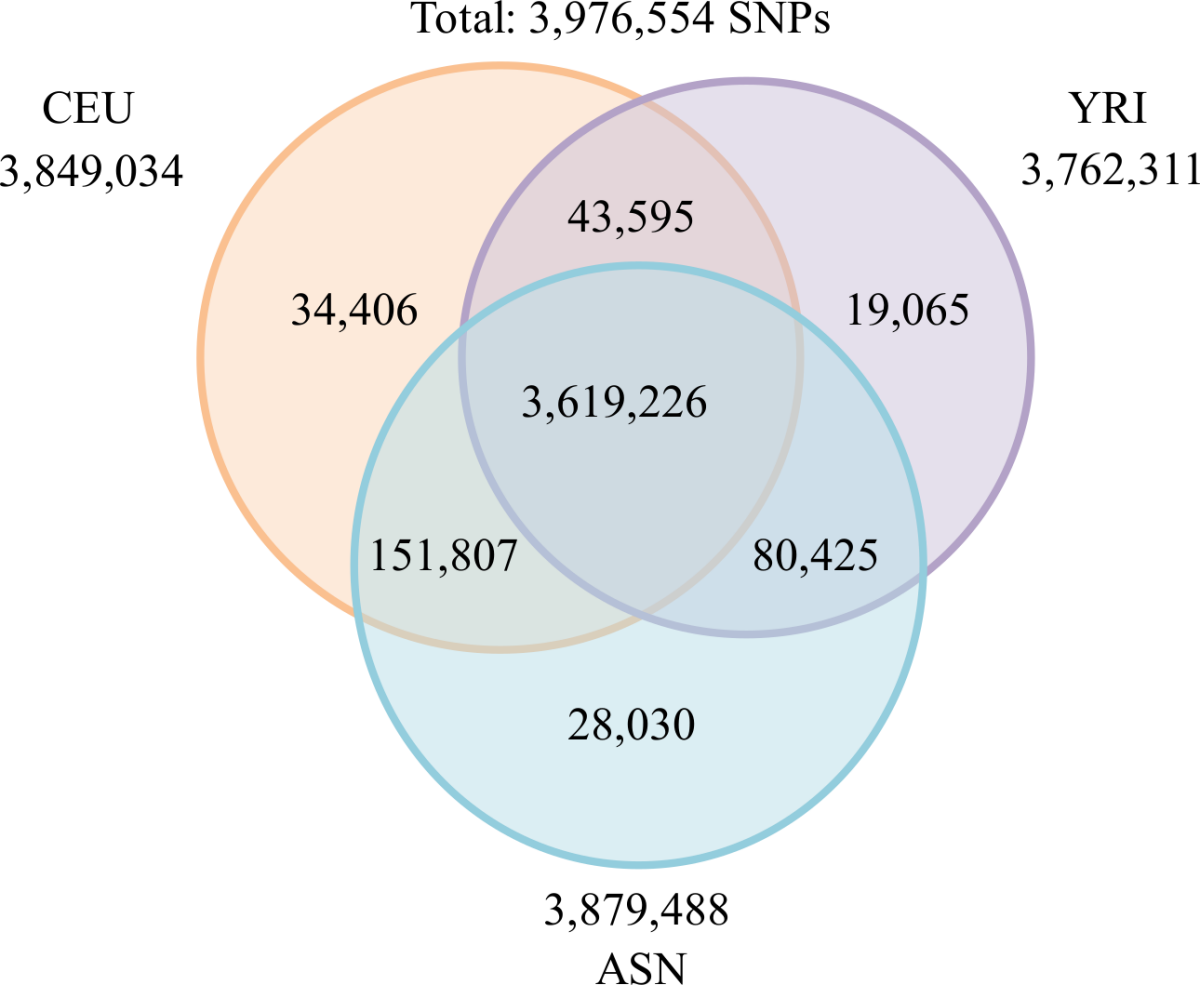
HapMap SNPs from three populations. The relationships between the numbers of SNPs in 22 autosomal chromosomes from three populations, YRI, CEU, and ASN, in the HapMap database are shown. A total of 3,619,226 SNPs were found in all three populations. Among them, 879,657 SNPs were selected under the condition that all of the SNPs could be attributed to the genotypes of all 270 individuals.

We downloaded unphased diplotype data sets of 22 autosomal chromosomes from release 24 of the HapMap database [35]. The data sets consisted of unphased diplotypes of 270 individuals: 90 Yoruba from Ibadan, Nigeria (YRI); 90 Utah residents with ancestry from northern and western Europe (CEU, from the CEPH diversity panel); and 90 Japanese from Tokyo and Japan, and Han Chinese from Beijing, China (ASN). All markers in the data set were diallelic. We selected 3,619,226 SNPs that were common to the three populations (Fig 2); among these, 879,657 SNPs had no missing data. The genotypes of these 879,657 SNPs were used to identify ancient haplotype blocks that were present in African populations and spread with migrating populations.

The Entrez SNP search tool (https://www.ncbi.nlm.nih.gov/snp) was used to retrieve nonsynonymous SNPs (nsSNPs) from dbSNP build 132. We downloaded all three kinds of nsSNPs: 173,911 missense, 6,838 nonsense, and 24,296 frame-shift SNPs, among which 4,316 nsSNPs were included in the HapMap data sets. CCDS [38] build 36.3 was further used to evaluate the location of each SNP in terms of protein-coding genes. In total, 3,298 nsSNPs were mapped to 2,467 genes across the 22 autosomal chromosomes.

### KEGG for functional annotation

KEGG is a suite of databases that includes molecular interaction networks (PATHWAY database) and information about genes and proteins (GENES/SSDB/KO databases), and biochemical compounds and reactions (COMPOUND/GLYCAN/REACTION databases) [36]. We used KEGG PATHWAY, which includes 430 reference pathway maps (downloaded on 25 February 2015), among which 74 are of human diseases. The human disease maps contain 12 cancer maps.

KEGG mapper is a web-based interface that accepts gene lists as input, and outputs lists of KEGG pathway maps that contain the genes in the input list. We used KEGG mapper to identify the functions of the genes obtained by our scans. We also used KEGG pathway maps for a Monte Carlo test that showed to which pathway maps the genes were likely to belong.

### Inter-diplotype distance

**Fig 3:**
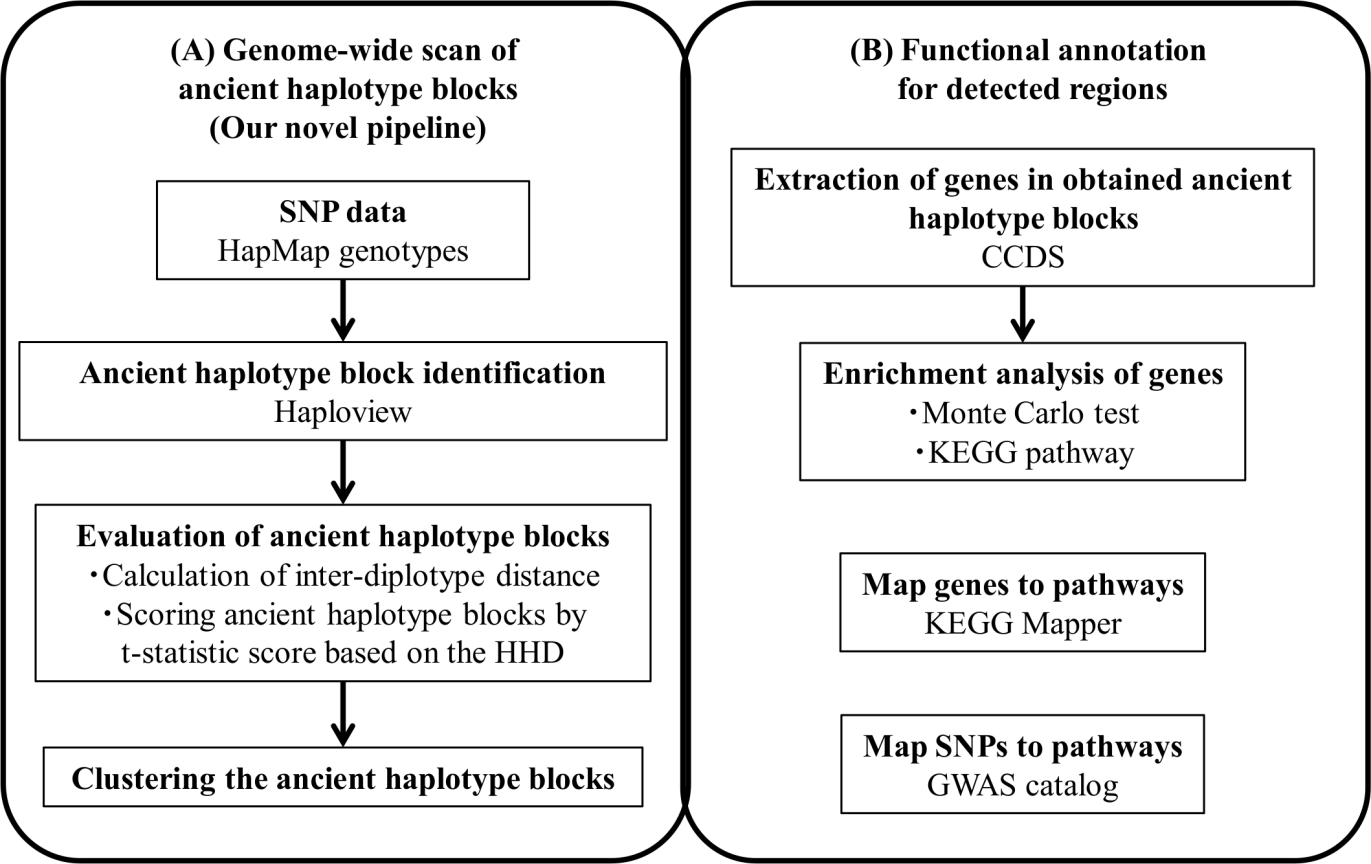
Pipeline for ancient haplotype block scan and functional annotation. (A) Novel procedure for ancient haplotype block scan using HHDs. (B) Functional annotation procedure based on biological pathways. Each box shows materials or tools used in that step.

In our previous work [39], we defined an inter-diplotype distance called Haplotype Inference Technique (HIT) Hidden Markov Model-based Distance (HHD). Unlike the allele sharing distance (ASD) [40], HHD reflects the founder (or ancestral) haplotypes well. HHD assumes multiple founder haplotypes [39] and calculates the distance between founder and present-day haplotypes. The distances between founder and present-day haplotypes were used to calculate the distance between individual SNP genotypes. If we hypothesize the existence of common founder haplotypes in several populations, HHD performs better than ASD. When specific haplotypes are conserved in populations, both HHD and ASD produce small values, but when they are not conserved, HHD produces much larger values than ASD. Thus, for blocks that have both common founder and population-specific haplotypes, it is highly possible that the inter-population HHD would be larger than ASD. Therefore, we implemented a pipeline that utilizes HHD (Fig 3).

Briefly, the difference between HHD and ASD in terms of their algorithms is as follows. The algorithm for ASD between genotypes first counts allele differences at each SNP site; then, the total allele differences are normalized. The HHD algorithm first infers candidate haplotypes and their frequencies in populations for each genotype. Second, it calculates distances between candidate haplotypes of two genotypes. The distances between candidate haplotypes are weighted by their frequencies in the populations. Finally, for HHD, the distances between candidate haplotypes are added and normalized. Unlike ASD, HHD identifies differences between common founder and present-day haplotypes. When haplotype composition of two populations are similar, HHD between the genotypes is small like ASD. If two populations have different haplotype composition, HHD calculates the distance between genotypes more accurately and becomes larger than ASD. If of the difference between average HHD values between two populations is large, we infer that the region has haplotype variation and it is possible that there are population-specific haplotypes.

### Genome-wide scan of ancient haplotype blocks (Fig 3A)

#### 1. Identification of ancient haplotype blocks

We assumed that functionally important conserved regions in African populations spread with other populations during human migration. Currently, such conserved regions differ by population but may have shared regions [41]. We defined the shared regions as ancient haplotype blocks.

We first identified haplotype blocks for each population with Haploview 4.2 [42]. Haploview estimates Hedrick’s multiallelic D′ [43, 44] between a pair of SNPs, and 95% confidence bounds on D′ are used to evaluate the strength of LD between the SNP pair. The default setting of Haploview ignores pair-wise comparisons of SNPs further than 500 kb apart.

Next, we extracted the haplotype blocks of the YRI population that overlapped with the haplotype blocks of both the CEU and ASN populations. For a haplotype, let *H*[*i*..*j*] denote the haplotype, where positions of the first and last SNPs are *i* (bp) and *j* (bp) in the genome. Two haplotypes, *H*1[*i*..*j*] and *H*2[*k*..*l*], are thought to overlap with each other in any of the following: *i* ≤ *k* ≤ *j* ≤ *l*, *k* ≤ *i* ≤ *l* ≤ *j*, *i* ≤ *k* ≤ *l* ≤ *j* or *k* ≤ *i* ≤ *j* ≤ *l*. We considered the extracted haplotype blocks of the YRI population as ancient positive selection candidates that spread with population migration.

To identify the shared regions of the haplotype blocks, we detected common haplotype blocks. Here, the common haplotype blocks were defined as the haplotype blocks obtained from genotype data of all three populations. To evaluate whether the identified common haplotype blocks were affected by ancient positive selection and really exist for each population, we further searched the common haplotype blocks that overlapped with the previously extracted candidates to identify ancient positive selection events. We defined the extracted final set of haplotype blocks as ancient haplotype blocks.

**Fig 4:**
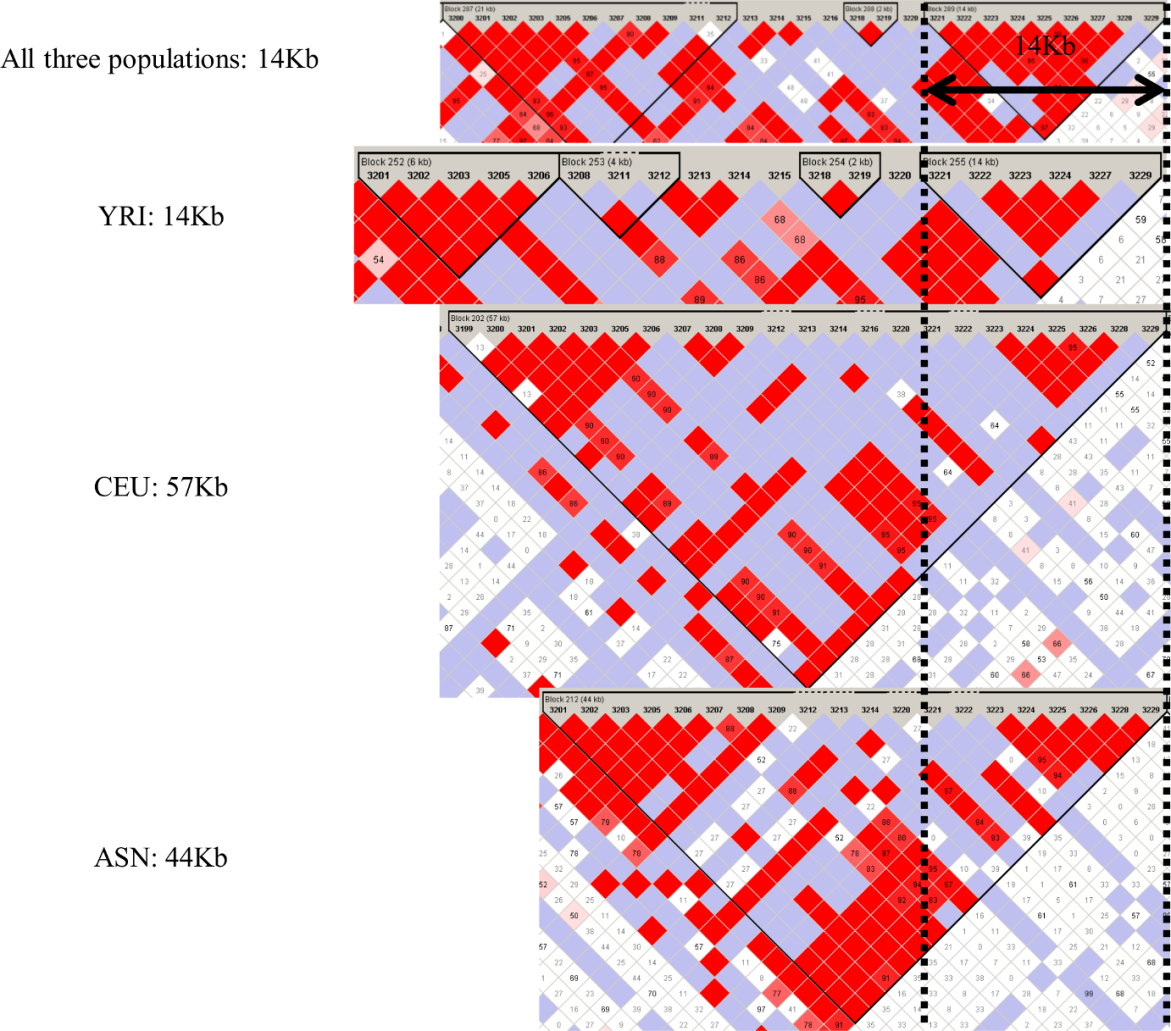
Example of ancient haplotype blocks identified in this work. Four haplotype blocks identified in all three populations (YRI, CEU, and ASN) are shown. The region of overlap between the dashed lines is defined as the ancient haplotype block.

Fig 4 shows an example of ancient haplotype blocks that were identified from the 879,657 genotypes. The 14-kb haplotype block was identified in 270 individuals, already existed in the YRI population, and overlapped with the haplotype blocks of the CEU and ASN populations. Although recent studies analyzed population-specific features of LD distribution [45], we identified haplotype blocks common to all of the populations for ancient haplotype block regions.

#### 2. Calculation of inter-population distances for ancient haplotype blocks

For the k-th ancient haplotype block, we calculated HHD between two individuals *i* and *j*, *d*_*ijk*_ (1 ≤ *i* < *j* ≤ 270), across all three populations and constructed a 270 × 270 HHD matrix for each ancient haplotype block (S1 Text, S1 Fig). To identify ancient haplotype blocks that differed between populations (i.e., ancient haplotype blocks with common founder haplotypes and population-specific haplotypes), we used a t-statistic score based on inter-population distance *X*_*k*_ and intra-population distance *Y*_*k*_ for each haplotype block *k*:

$$t_{k}=\frac{\bar{X_{k}}-\bar{Y_{k}}}{\sqrt{s_{{XY}_{k}}(\frac{1}{m}+\frac{1}{n})}},$$ (1)

where

$$s_{{XY}_{k}}=\frac{(m-1)s_{X_{k}}+(n-1)s_{Y_{k}}}{m+n-2},$$

*m* is the total number of inter-population pairs of individuals that belong to different populations, and *n* is the total number of intra-population pairs of individuals that belong to the same population (S1 Fig). $\bar{X_{k}}$ and $\bar{Y_{k}}$ are the sample means of the inter- and intra-population distances, and $s_{X_{k}}$ and $s_{Y_{k}}$ are the unbiased variances of the inter- and intra-population distances. This score measures the difference between the mean HHD value for pairs of people that belong to different populations (inter-population distance) and pairs of people that belong to the same population (intra-population distance); if the score is high, the haplotype block is considered to represent a difference between populations. We ranked the ancient haplotype blocks with this score for the three populations. We considered that blocks in the upper tail of the score distribution (i.e., top 1% of blocks) were likely to have common founder and population-specific haplotypes that were created by ancient positive selection and population-specific mutations. In the present work, top 1% of blocks were considered to show population differentiations and further validated by the following steps (see “Relationship between the top 1% of blocks and Fst” for additional detail).

#### 3. Ancient haplotype block characterization

We used networks that represented differences between the three populations evaluated using t-statistic scores (Fig 1B) to classify the ancient haplotype blocks. Each node of the network represented a population (i.e., YRI, CEU or ASN), and the weight of each edge represented the sample mean of t-statistic scores between the two populations. *k*-means clustering was applied to all the ancient haplotype blocks based on the weights of the three edges, CEU–YRI, CEU–ASN, and ASN–YRI.

### Functional annotation of candidate regions (Fig 3B)

#### 1. Monte Carlo test for enrichment analysis

We performed KEGG pathway enrichment analysis using the genes in the detected ancient haplotype blocks, and evaluated the result by Monte Carlo test using the genes obtained from 10,000 random samples of 310 ancient haplotype blocks (1% of all ancient haplotype blocks). The Jaccard index was used as a measure of the overlap between all genes in a KEGG pathway and the genes in the ancient haplotype blocks. For each pathway, p-values were calculated based on the distribution of the Jaccard index of random samples.

#### 2. Annotation of genes and SNPs by pathway mapping and GWAS catalog

We mapped genes in the detected regions to biological pathways in the KEGG database. We also investigated known phenotypes associated with SNPs in the regions using the NHGRI GWAS catalog [37], which collects relationships between SNPs and human phenotypes. The SNPs that have known phenotypes were then mapped to biological pathways through reported genes. KEGG Mapper was used to identify associated biological pathways and their functional categories.

## Results

### Identification of ancient haplotype blocks

In the 22 autosomal chromosomes, Haploview [42] identified 62,123, 56,597, and 56,325 haplotype blocks in the YRI, CEU, and ASN populations, respectively. We also identified 76,119 haplotype blocks in all three populations, 39,228 of which were defined as ancient haplotype blocks. Of these, we used 30,966 ancient haplotype blocks that consisted of more than two SNPs. The maximum, minimum, and average lengths of the identified ancient haplotype blocks were 499,794, 42, and 24,584.36 bp, respectively. The average length of 24,584.36 bp is much shorter than that of the regions identified by studies based on previous LD-based methods, such as the long-range haplotype test [27, 28], which focuses on recent positive selection (Table 1). The number of SNPs and genes in the blocks varied from 3 to 97 and 0 to 6, respectively. The total number of SNPs and genes in the identified ancient haplotype blocks were 240,752 and 5,577, respectively.

**Table 1:** Average length of regions identified by representative methods.

Method	Average lengths (bp)
LRH, iHS [21]	310,049.59
LRH, iHS, XP-EHH [22]	151,579.03
EHHS [23]	336,811.55
CMS [24]	86,178.84
XP-CLR [25]	1,280,084.33
HaploPS [26]	449,043.75
Ancient haplotype blocks by the present study	24,584.36
Top 1% t-score of the ancient haplotype blocks	35,803.89

### Inter-population distances

**Fig 5:**
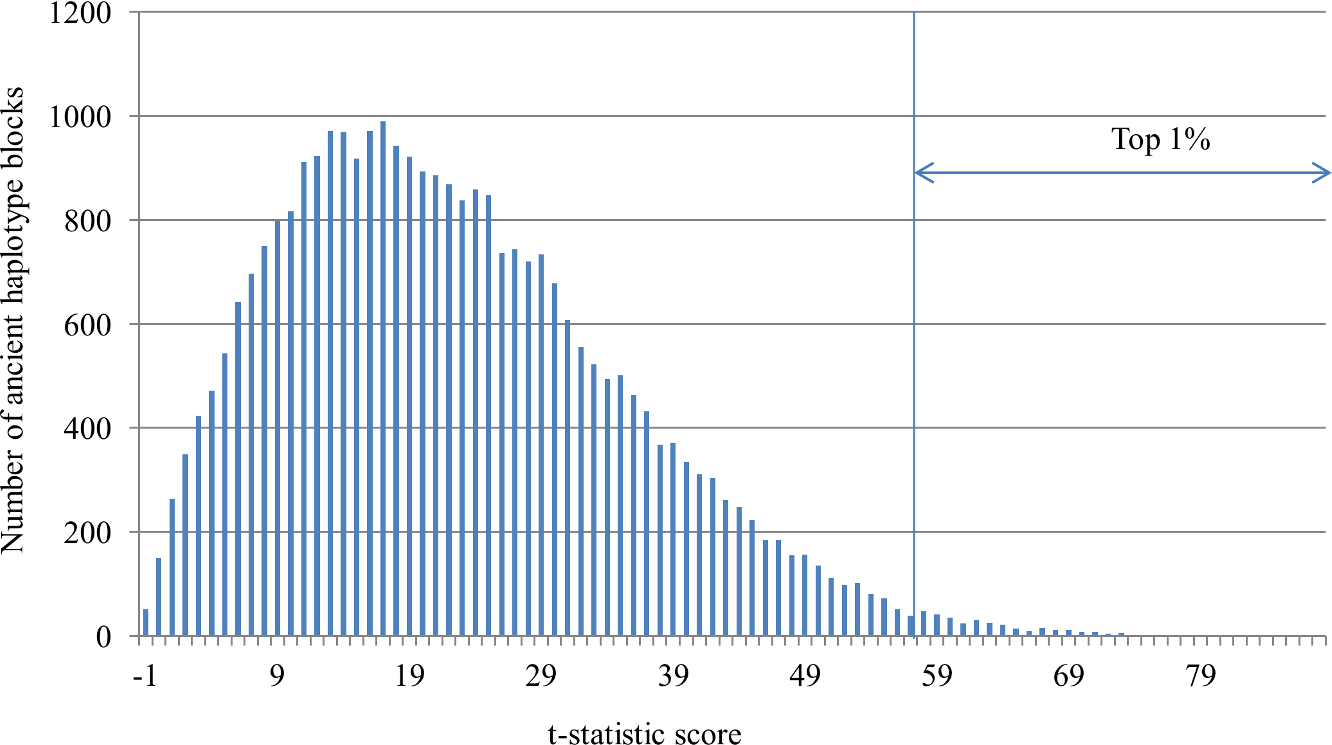
Distribution of calculated scores. The x-axis shows the t-statistic score, and the y-axis shows the number of ancient haplotype blocks.

To find haplotype blocks that represent differences among the three populations, we calculated the t-statistic score, *t*_*k*_, which was defined in Eq (1), for each ancient haplotype block. Fig 5 shows the distribution of the calculated scores. The distribution can be fitted to the generalized extreme value (GEV) distribution. Larger scores represent greater disparity between inter-population and intra-population distances. In the top 5% of sorted haplotype blocks, there was a set of 1,548 haplotype blocks that includes 592 genes and 13,955 SNPs. When we examined the top 1% of sorted haplotype blocks, we identified a set of 310 haplotype blocks. The 310 haplotype blocks included 130 genes (S1 Table, S2 Table) and 2,803 SNPs. The average length of the 310 ancient haplotype blocks was 35,803.89 bp (Table 1). Additionally, 35% and 49% of the SNPs had Fst [2] values larger than 0.2 in the top 5% and 1% of blocks, respectively. The average Fst values for the SNPs in the top 5% and 1% of blocks are 0.162 and 0.187, which are significantly different based on the two-tailed Welch’s t-test (*p*-value < 0.05). (see “Relationship between the top 1% of blocks and Fst” for additional detail).

### Characterization of ancient haplotype blocks

**Fig 6:**
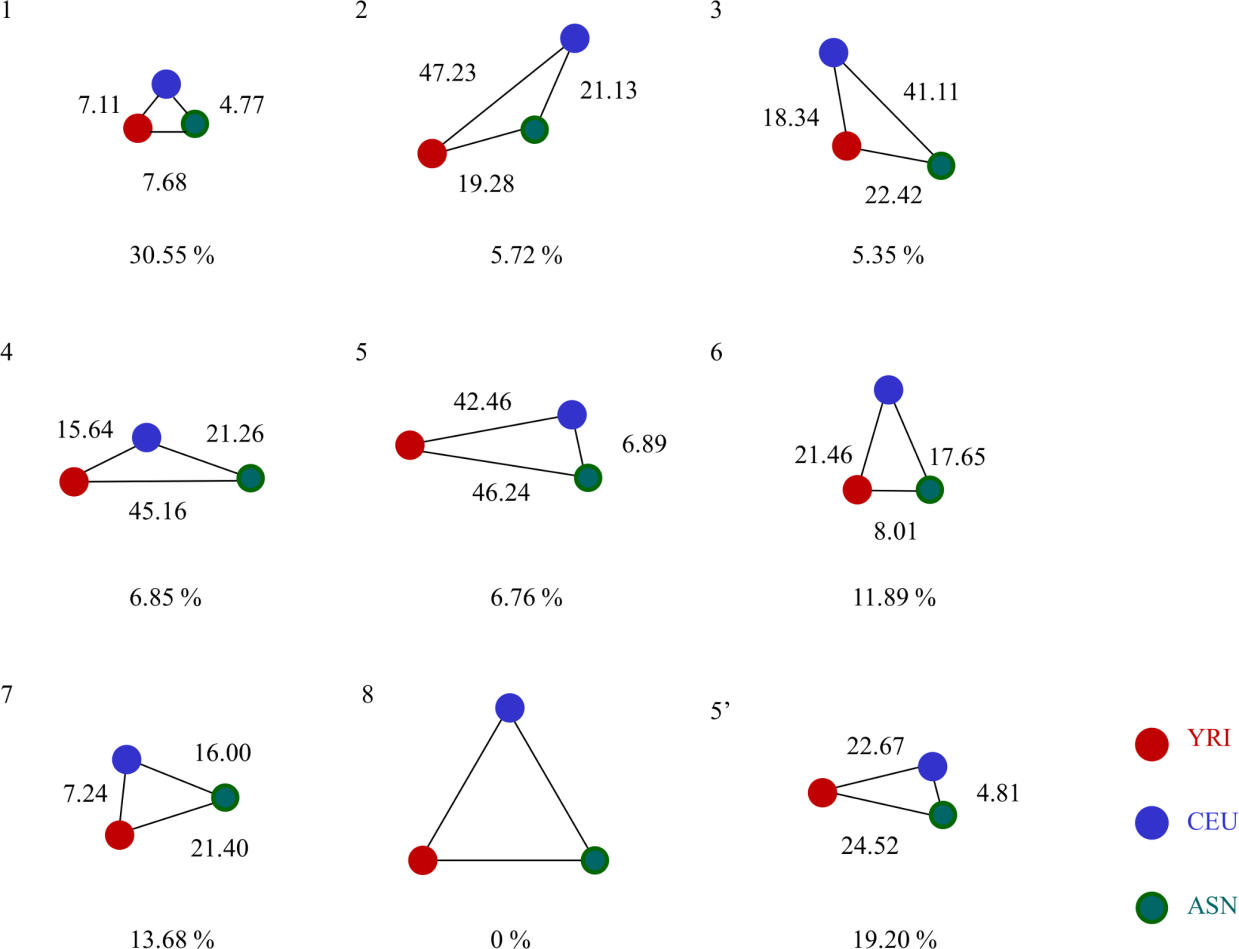
Classification of ancient haplotype blocks. Eight clusters of ancient haplotype blocks obtained by clustering based on the network of populations and their t-statistic score profiles. The number on each edge represents the average t-statistic score; smaller scores reflect shorter edges.

We classified all ancient haplotype blocks into eight clusters (i.e., *k* = 8 for *k*-means clustering) based on the network of populations and their t-statistic score profiles (Fig 6, S3 Table). We used *k* = 8, because the network with three edges can be classified into eight patterns if we classify each edge as either long or short. Using this setting, we could not find Cluster 8 that corresponds to a network with all three edges long. Instead, Cluster 5′, which was similar to Cluster 5, was obtained. However, the degrees of the differences for the YRI population pairs were much smaller for Cluster 5′. The largest portion (~30%) of the ancient haplotype blocks was classified in Cluster 1 (Table 2). Clusters 2, 3, 4, and 5 had almost the same number of cluster members. Clusters 6 and 7 had almost twice as many cluster members as Clusters 2, 3, 4 and 5.

**Table 2:** Summary of screening results.

Cluster	1	2	3	4	5	6	7	5’	Total
Top 1%	0	76	39	35	160	0	0	0	310
	(0%)	(24.52%)	(12.58%)	(11.29%)	(51.61%)	(0%)	(0%)	(0%)	
Total	9,459	1,772	1,657	2,121	2,094	3,682	4,237	5,944	30,966
	(30.55%)	(5.72%)	(5.35%)	(6.85%)	(6.76%)	(11.89%)	(13.68%)	(19.20%)	

Each element in the table shows the number of obtained haplotype blocks. The numbers in parentheses are percentages of the total pool of haplotype blocks.

### Association between clustering results and t-statistic score

**Fig 7:**
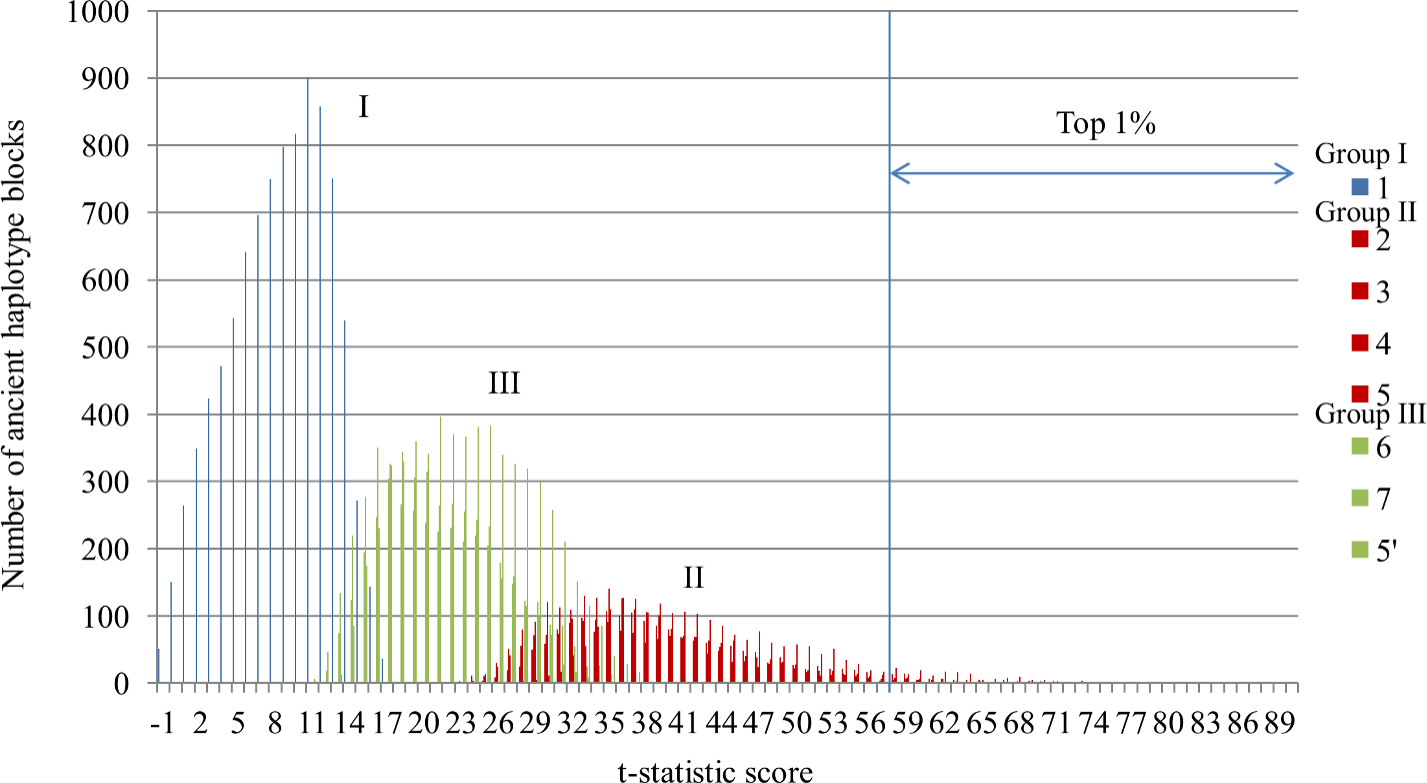
Score distributions for each cluster. The score distribution of ancient haplotype blocks is shown for each cluster. The clusters can be classified into three groups: I, II, and III. Group I consists of Cluster 1 (blue). Group II consists of Clusters 2, 3, 4, and 5 (red). Group III consists of Clusters 6, 7, and 5′ (green).

Based on the score distribution for each cluster shown in Fig 5, the clusters can be classified into three groups: group I, which consists of Cluster 1; group II, which consists of Clusters 2, 3, 4, and 5; and group III, which consists of Clusters 6, 7, and 5′ (Fig 7). The largest portion of the ancient haplotype blocks was classified in group I, with scores below 18, and showed no large differences across the three populations. The scores of groups III and II ranged from 11 to 39 and 23 to 86, respectively.

The top 1% of the sorted ancient haplotype blocks contained significantly higher proportions of Clusters 2 and 5 than the total pool of ancient haplotype blocks (*p*-value < 0.05) (Table 2). This result for Cluster 5 is consistent with the previous results, which indicates that the genetic distance between the African population and the other populations is large [46, 47]. Our results also showed that twice as many members of Cluster 2 are in the top 1% that of Cluster 4.

### Functional annotation of blocks in the top 1% of t-statistic scores

The Monte Carlo test for enrichment of genes in the top 1% of ancient haplotype blocks (310 haplotype blocks) showed that the 130 genes were enriched for 22 pathways categorized in “Metabolism,” “Genetic Information Processing,” “Cellular Processes,” “Organismal Systems,” and “Human Diseases” (Table 3). In the “Human Diseases” pathways, we found several diseases already known to have some differences between populations: hepatitis C, non-alcoholic fatty liver disease (NAFLD), and some cancers.

**Table 3:** Pathways for which the genes in the top 1% of ancient haplotype blocks are enriched.

Category	Pathway	Genes*				p-value
		Cluster 2	Cluster 3	Cluster 4	Cluster 5	
Organismal Systems	T cell receptor signaling pathway	GSK3B	IL10,		PAK7	0.029
Immune system						
Nervous system	Neurotrophin signaling pathway	GSK3B,	SH2B3		BRAF, RPS6KA2	0.007
Endocrine system	Progesterone-mediated oocyte maturation				BRAF, GNAI1, MAD1L1 RPS6KA2	0.005
Metabolism	beta-Alanine metabolism	GADL1		ACADM		0.016
Metabolism of other amino acids						
Genetic Information Processing	Ribosome biogenesis in eukaryotes				EFTUD1, RBM28	0.039
Translation						
Environmental Information Processing	Neuroactive ligand receptor interaction	GLP2R,	ADRA1A, CHRNB4,		PARD3 GRID2 GRIK1 GRIK2,	0.012
Signaling molecules and interaction						
Signal transduction	Hippo signaling pathway	GSK3B			APC, DLG2, PARD3	0.048
Cellular Processes	Focal adhesion	GSK3B, LAMA3 MYLK	ACTN1,		BRAF, PAK7	0.018
Cellular community						
	Signaling pathways regulating pluripotency of stem cells	GSK3B,			APC, JAK1	0.019
	Tight junction		ACTN1, JAM2,		GNAI1, PARD3, PRKCH	0.038
Cell motility	Regulation of actin cytoskeleton	MYLK,	ACTN1,		APC, BRAF, PAK7, PIP5K1B SSH2	0.001
Human Diseases	Toxoplasmosis	LAMA3	IL10,		GNAI1, JAK1,	0.003
Infectious diseases						
	Hepatitis C	GSK3B,			BRAF, JAK1	0.022
	Pertussis		IL10		GNAI1,	0.023
	Leishmaniasis		IL10,		JAK1	0.025
Cancers	Colorectal cancer	GSK3B			APC, BRAF, DCC,	0.001
	Renal cell carcinoma				ARNT2, BRAF, PAK7	0.008
	Endometrial cancer	GSK3B			APC, BRAF,	0.018
	Basal cell carcinoma	GSK3B			APC,	0.023
	Viral carcinogenesis		ACTN1,		JAK1, MAD1L1	0.046
Endocrine and metabolic diseases	Non-alcoholic fatty liver disease (NAFLD)	GSK3B NDUFS6			NDUFA8	0.016
Neurodegenerative diseases	Parkinson's disease	NDUFS6			GNAI1, NDUFA8,	0.013

* Enriched genes in each cluster.

Hepatitis C varies (HCV) in incidence rate and treatment response across populations [48]. The chronic HCV infection rate is higher in African Americans than in people of European ancestry in the United States. It has also been reported that histologic progression of HCV infection is less rapid among African American patients than among those of European ancestry. Rates of adverse events are higher among patients of European ancestry. The rate of sustained virologic response in African Americans is significantly lower than for patients of European ancestry. In our results, BRAF (Cluster 5), GSK3B (Cluster 2), and JAK1 (Cluster 5) were mapped to “Hepatitis C.” BRAF and JAK1 have not previously been found to be affected by positive selection, but GSK3B was reported to be affected by positive selection in people of Mexican ancestry in Los Angeles, California, USA [26].

Differences in HCV-specific CD4 T cell responses between African Americans and people of European ancestry have been previously discussed, and may explain some of these differences across populations [48]. Previous haplotype analyses have also suggested that variants of the immunomodulatory IL10 and IL19/20 genes play a role in the spontaneous clearance of HCV in African American patients but not in patients of European ancestry [49]. The “T cell receptor signaling pathway” appeared in our results, and IL10 (Cluster 3) GSK3B (Cluster 2) and PAK7 (Cluster 5) were mapped to this pathway.

NAFLD, an endocrine and metabolic disease, has been suggested to have pathophysiological differences among populations [50]. Latinos (45%) show the highest prevalence of hepatic steatosis and African Americans show the lowest prevalence; people of European ancestry showed an intermediate prevalence of 33% [50]. There might be differences in metabolic responses related to NAFLD in different populations. NDUFA8 (Cluster 5), NDUFS6, and GSK3B (Cluster 2) were mapped to “Non-alcoholic fatty liver disease (NAFLD)”. NDUFA8 has been reported to be affected by positive selection in European populations [23], but NDUFS6 has not previously been found to be affected by positive selection.

Regarding cancers, higher renal cell carcinoma incidence rates have been identified in men of African ancestry [51]. Endometrial cancer is reported to have higher incidence rates in women of European ancestry than in any other population [52, 53]. Basal cell carcinoma is known to be common in fair-skinned individuals [54]. ARNT2 (Cluster 5), BRAF (Cluster 5), and PAK7 (Cluster 5) were mapped to “Renal cell carcinoma;” APC (Cluster 5), BRAF (Cluster 5), and GSK3B (Cluster 2) were mapped to “Endometrial cancer;” and APC (Cluster 5) and GSK3B (Cluster 2) were mapped to “Basal cell carcinoma” in our results. APC has been reported to be a positive selection candidate in European and Asian populations [24, 26], and the others have not previously been reported to be affected by positive selection.

### Functional annotation of genes and SNPs in each cluster

To check the functional annotation details of the top 1% of regions, which included only members of Clusters 2, 3, 4, and 5, as previously discussed, we mapped the genes and SNPs in each cluster to pathways and the GWAS catalog, respectively.

#### Cluster 2

The 76 ancient haplotype blocks in Cluster 2 included 34 genes (S2 Table). Nine genes had previously been reported as being affected by positive selection (S4 Table) [21, 23–26]. ARHGAP30 and USF1 in Cluster 2 have been reported to show especially strong signals of positive selection in African populations [24].

Ten genes were mapped to 58 pathway maps (i.e., five “Metabolism”, nine “Environmental Information Processing,” five “Cellular Processes,” 21 “Organismal Systems,” and 18 “Human Diseases” pathways. In addition to the pathways that appeared in the enrichment analysis, GSK3B was mapped to the “Immune System” pathways “B cell receptor signaling pathway” and “Chemokine signaling pathway,” and MYLK was mapped to “Platelet receptor signaling pathway.” Regarding infectious diseases, GSK3B was mapped to “Amoebiasis,” “Epstein–Barr virus infection,” “HTLV-I infection,” “Influenza A,” and “Measles.”

In the NHGRI GWAS catalog, five SNPs in 76 haplotype blocks were previously reported [55–58]. These five SNPs in Cluster 2 were associated with bone mineral density, prostate-specific antigen levels, hair morphology, and breast cancer (S5 Table). Only one SNP, rs9383951, which was associated with breast cancer, was mapped to a KEGG pathway through ESR1.

#### Cluster 3

The ancient haplotype blocks in Cluster 3 included 17 genes (S2 Table). Eight were previously reported as candidates of positive selection (S4 Table) [26]. SH2B, known to be associated with celiac disease, is in Cluster 3 and has been reported to be under convergent evolution in Asia and Europe [26].

Eight genes were mapped to 40 pathway maps, which included one “Genetic Information Processing,” eight “Environmental Information Processing,” five “Cellular Processes,” eight “Organismal Systems,” and 18 “Human Diseases” pathways. In addition to the pathways that appeared in the enrichment analysis, IL10 was mapped to immune system-related pathways such as the “Jak-STAT signaling pathway,” and immune system-related diseases such as “Asthma,” “Inflammatory bowel disease (IBD),” “Systemic lupus erythematosus,” “Epstein–Barr virus infection,” and “Malaria.” IL10 has been reported to be associated with pathogen diversity and susceptibility to autoimmune diseases [17].

In the NHGRI GWAS catalog, two SNPs in 39 haplotype blocks were previously reported [59, 60]. We found two SNPs, rs1194289 and rs7101446, in Cluster 3 associated with response to anti-depressant treatment in major depressive disorder, and economic and political preferences (S5 Table). These two SNPs were not mapped to any KEGG pathways.

#### Cluster 4

The ancient haplotype blocks in Cluster 4 included nine genes (S2 Table). Two, ACADM and EML4, were previously reported to be affected by positive selection in Asian populations (S4 Table) [23, 26].

There were no immune system-related genes in Cluster 4. However, there were some genes related to metabolism. Two genes, ACADM and EML4, were mapped to six pathways, four of which were metabolism pathways (S6 Table).

In the NHGRI GWAS catalog, we found only one SNP, rs4949874, in 35 haplotype blocks that were previously reported [61]. We found that this SNP is associated with blood metabolite ratio and mapped to four metabolism pathways through the ACADM gene (S5 Table).

#### Cluster 5

The 160 ancient haplotype blocks in Cluster 5 included 70 genes (S2 Table). Fourteen genes were positive selection targets (S4 Table).

Thirty genes were mapped to 109 pathway maps, which included 12 “Metabolism,” two “Genetic Information Processing,” 17 “Environmental Information Processing,” nine “Cellular Processes,” 28 “Organismal Systems,” and 41 “Human Diseases” pathways. JAK1 was mapped to some immune system-related pathways such as “Jak-STAT signaling pathway” and immune system-related disease pathways such as “Epstein–Barr virus infection” and “Hepatitis C.”

In the NHGRI GWAS catalog, 12 SNPs in 160 haplotype blocks were previously reported [62–72]. Among them, we found that SNP rs10056340 in Cluster 5 was associated with “Allergic sensitization” in the European population (S5 Table).

SNP rs10056340 is in low LD with six nearby variants (rs17513503, rs1837253, rs3806932, rs1898671, rs2416257, and rs2416257) in people of European ancestry [69]. These variants were reported to be associated with eosinophil counts and atopic asthma (rs2416257), pediatric eosinophilic esophagitis (rs3806932), asthma (rs1837253, rs1438673), and allergic rhinitis (rs17513503 and rs1898671). SNP rs10056340 is considered to represent a new causal variant for allergic disease in this region [69]. Four genes (SLC25A46, TSLP, WDR36, and CAMK4) are near or in the LD region that contains rs10056340. SNP rs10056340 is associated with CAMK4 expression in lymphoblastoid cell lines. CAMK4 was also previously reported to be a target of positive selection in the European population [23].

Among these four genes, TSLP, WDR36, and CAMK4 were mapped to 12 pathways, including immune system-related pathways such as “Jak-STAT signaling pathway” and “Cytokine–cytokine receptor interaction.” The other genes mapped to these pathways are IL10 (Cluster 3) and JAK1 (Cluster 5). Both IL10 and JAK1 are mapped near the receptors in the “Jak-STAT signaling pathway.” These genes were also mapped to “Epstein–Barr virus infection” pathway as members of the “Jak-STAT signaling pathway.”

## Discussion

In this work, we proposed a novel LD-based pipeline to identify ancient positive selection events from SNP data by hypothesizing that regions positively selected in ancient times contain important functions in the immune system and create variations in common diseases among populations. Based on this framework, we first identified ancient haplotype blocks, and then scanned the identified ancient haplotype blocks to check for haplotype frequency variation among populations. For the scans of ancient haplotype blocks, we used a measure, HHD, that employs differences in haplotype frequencies among populations.

### Ancient haplotype block features

By applying our pipeline to HapMap2 genotypes, we found that a large portion of the ancient haplotype blocks showed no large differences in LD patterns among the three populations, and 75.32% of the ancient haplotype blocks were in Groups I and III (Figs 5 and 7). Our scan revealed that many of the ancient haplotype blocks that showed large differences among the three populations were regions that have YRI-specific haplotypes (Cluster 5). This is consistent with the fact that YRI populations are the most distant from the other populations based on phylogenetic tree analyses [46, 73]. However, we also detected ancient haplotype blocks that showed larger differences between YRI and CEU populations than between YRI and ASN populations (higher scoring blocks in Cluster 2 than Cluster 4).

Previously inferred phylogenetic trees of human populations have shown that YRI and CEU populations are more closely related than YRI and ASN populations [46, 73]. The result of YRI and CEU showing large differences may be specific to ancient haplotype blocks. We need to further examine these results. We also performed functional analyses of extracted haplotype blocks and clusters, and we discuss the results in detail in the following subsections.

### Relationship between the top 1% of blocks and Fst

We used Fst to check whether our pipeline detected positive selections, because positive selections create large allele frequency differences, and Fst measures allele frequency differences between populations. SNPs affected by positive selection tend to accumulate in the top tail of Fst distribution [2].

Our result showed that the average value of Fst for the SNPs in the top 1% of blocks was significantly larger than that of the top 5% of blocks (*p*-value < 0.05), which indicates that the top 1% of blocks included more positive selection candidate SNPs. However, in the top 0.5% of blocks, 54% of the SNPs had Fst values greater than 0.2. We considered there to be little difference between the top 0.5% and top 1% of blocks based on Fst values greater than 0.2. Therefore, we chose the top 1% of blocks for the present study.

Although Fst measures population differentiations based on allele frequencies, our score measures population differentiation based on haplotype frequencies. By focusing on the ancient haplotype blocks, we tailored our pipeline to detect SNPs in functionally important haplotype blocks among SNPs with large Fst values. Functions of SNPs and genes detected through our pipeline are discussed in the following sections.

### Genes in the top 1% of blocks enriched in immune system-related pathways

In the top 1% of the sorted ancient haplotype blocks, we found genes enriched in immune system-related pathways and immune system-related disease pathways. We found genes enriched in immune system pathways such as “T cell receptor signaling pathway,” infectious disease pathways such as “Hepatitis C,” and endocrine and metabolic disease pathways such as “Non-alcoholic fatty liver disease (NAFLD).” It is possible that the genes mapped to these pathways may be associated with the differences between these diseases.

The genes mapped to “Hepatitis C” pathway were identified as having been affected by ancient selection and associated with differences in incidence rate and treatment response between the African population and other populations. GSK3B in particular is suggested to be associated with differences between African and European populations. It may be interesting to examine the association between the genes mapped to this pathway, such as IL10, and hepatitis C in future analyses.

Additionally, IL10, an anti-inflammatory cytokine, maps to “T cell receptor signaling pathway” and the infectious diseases “Toxoplasmosis,” “Pertussis,” and “Leishmania.” IL10 is a malaria-related gene [74–77]. IL10 may be affected by ancient positive selection in Africa and may now affect differences in these infectious diseases through the T cell receptor signaling pathway.

Among the genes mapped to “Non-alcoholic fatty liver disease (NAFLD)”, the two genes in Cluster 2 may be associated with differences in metabolic response between African and European populations. Ancient selection in NDUFA8 may be associated with the low prevalence of hepatic steatosis in African Americans [50].

Regarding cancers, the mapped genes in Cluster 5 may be affected by ancient positive selection associated with differences in cancer incidence rates between people of African ancestry and other populations. GSK3B in Cluster 2 may be affected by ancient positive selection and positive selection in the European population, which may be especially associated with incidence rate differences of endometrial cancer and basal cell carcinoma between African and European populations [52–54].

### Historical context of genes and SNPs mapped to the pathways related to known phenotypic variations

The genes and SNPs in the top 1% of regions were also shown to map to immune system pathways such as “Jak-STAT signaling pathway” and “Cytokine–cytokine receptor interaction;” common diseases such as “Prostate cancer,” “Endometrial cancer,” “Renal cell carcinoma,” and “Basal cell carcinoma;” immune-system-related diseases such as “Asthma” and “Inflammatory bowel disease;” and infectious diseases such as “Epstein–Barr virus infection.” These diseases vary in incidence rates among populations. The functions of the genes and SNPs mapped to these pathways may be associated with disease incidence variation.

**Fig 8:**
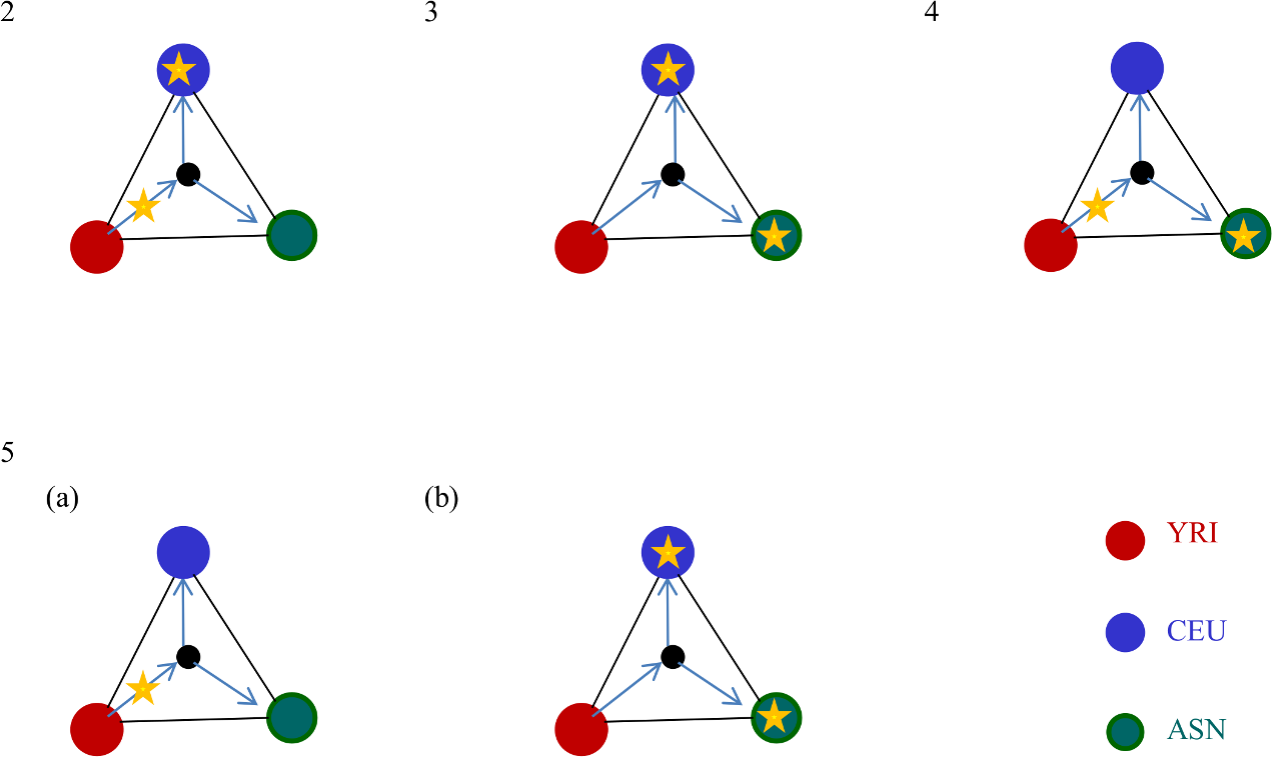
Assumed scenarios for the clusters in the top 1% of blocks. Each node represents a population, and each edge represents the degree of the t-statistic score between two populations. Red, blue, and green nodes represent YRI, CEU, and ASN populations, respectively. Asterisks represent mutations. The mutations were assumed to occur during or after migration, and are represented by asterisks on the arrows or the edges, respectively.

For these genes and SNPs in the top 1% of blocks that were mapped to the pathways, we discussed the possibilities of positive selections for each cluster based on its each historical scenario summarized in Fig 8. Because the average score between YRI and CEU is higher than the other two pairs in Cluster 2 (Fig 6), high-scoring regions may show positive selection signatures in CEU in addition to YRI. Similarly, that of Cluster 3 may show signatures of positive selection in both the CEU and ASN populations in addition to YRI. Cluster 4 may show selection in the YRI and ASN populations. Cluster 5 shows more differences between YRI and the other two populations than between CEU and ASN in the ancient haplotype blocks.

The genes in Cluster 2 are assumed to be affected by selection events in Africa and Europe (Fig 8). The immune system-related pathways where the genes in Cluster 2 were mapped may have been affected by ancient selection, and associated with differences in incidence rates of the diseases between YRI and CEU populations. The SNP mapped to the pathways through ESR1 may also be associated with variations in breast cancer between YRI and CEU populations. The genes in Cluster 3 are assumed to be affected by selection in Europe and Asia (Fig 8). Our results indicate that IL10 was affected by positive selection in ancient times and has different haplotype frequencies between CEU and ASN populations. In Cluster 4, there was a gene related to metabolism pathways. Our network model also explains the genes and SNPs in Cluster 4 as being affected by ancient selection and selection in Asia (Fig 8). For the network of Cluster 5, we consider two patterns, (a) and (b), based on when the mutations were introduced (Fig 8). The functions of the genes mapped to the immune system-related pathways through the GWAS catalog may be associated with differences in allergic sensitization between populations.

### Future work

In future analyses, we need to use more varied genome annotation information. In this analysis, we only used genes where SNPs existed for functional annotation. For example, we should check SNPs within 1 Mb of genes. We should also check coding regions, noncoding RNA genes, tRNAs, rRNAs, and microRNAs in future analyses.

Furthermore, we should improve our pipeline, especially the step for identifying ancient haplotype blocks, to produce more accurate identification and so that much larger data sets can be used. The scoring step should also be discussed so other statistical models can be introduced. Additionally, we will apply our pipeline to the genomes of species used as food, which will advance our understanding of human history.

## Supporting information

S1 Text Method for calculating HHD. Previously, we proposed a new measure between two genotypes called HHD. This is a brief explanation of how to calculate HHD.
(DOCX)

S1 Fig Inter- and intra-population distances. The inter- and intra-population distances in the HHD matrices used to calculate t-statistic scores.
(TIF)

S1 Table Candidate regions that contained genes in the HapMap2 data. The regions are listed by the t-statistic score assigned by our pipeline. Only the regions that contained genes are shown.
(DOCX)

S2 Table Genes in each cluster for the top 1% of regions. The genes are listed by cluster.
(DOCX)

S3 Table T-statistic score profiles of ancient haplotype blocks used for clustering. The t-statistic score profiles used for clustering are shown in the second, third, and fourth columns; each was calculated by pair-wise comparisons of populations. The fifth column shows the cluster number assigned by clustering. The sixth column shows the t-statistic score calculated for the three populations. The last column shows the genes in each haplotype block. The ID of each ancient haplotype block is the rs number of the first SNP in the block. Only the top 1% of ancient haplotype blocks that contained genes are shown here.
(DOCX)

S4 Table Genes previously reported to be affected by positive selection in each cluster. Genes that were previously reported to be affected by positive selection in the top 1% of ancient haplotype blocks are shown for each cluster. The genes are also classified according to the population where the positive selection was detected.
(DOCX)

S5 Table SNPs previously reported to be associated with some phenotypes by GWAS. SNPs in the top 1% of the ancient haplotype blocks that were previously reported [55–72] to be associated with phenotypes by GWAS are listed for each cluster. Each SNP is reported with the genes that the SNP is located in or linked with. The SNPs are mapped to the biological pathways through the reported genes. In addition to the reported genes, genes mapped to the biological pathways in the top 1% of ancient haplotype blocks were also listed in the sixth column.
(DOCX)

S6 Table Pathways where the Cluster 4 genes were mapped. There were no immune system-related genes in Cluster 4. However, there were some genes related to metabolism.
(DOCX)

## Abbreviations

SNP: single nucleotide polymorphism
nsSNP: nonsynonymous single nucleotide polymorphism
GWAS: genome-wide association study
LD: linkage disequilibrium
HHD: haplotype inference technique HHM-based distance
ASN: Japanese in Tokyo, Japan, and Han Chinese in Beijing, China
CEU: Utah residents with ancestry from northern and western Europe
YRI: Yoruba in Ibadan
KEGG: Kyoto Encyclopedia of Genes and Genomes
