# Supplementary material for: Revealing phenotype-associated functional differences by genome-wide scan of ancient haplotype blocks
Source: PLoS One. 2017 Apr 26;12(4):e0176530. doi: 10.1371/journal.pone.0176530 (PMC5406033; doi:10.1371/journal.pone.0176530)
Supplement: S1 Text — Previously, we proposed a new measure between two genotypes called HHD. This is a brief explanation of how to calculate HHD. (DOCX) [file pone.0176530.s001.docx]

For a haplotype $h\in S^{m}$ (where $S^{m}$ is a set of strings of length of $m$), let $h\left[ k \right]$ denote the allele of SNP at the *k*-th locus of $h$. The Hamming distance between two haplotypes $h$ and $h^{'}$ is defined as

$$s\left( h, h^{'} \right)= \sum_{k=1}^{m} I\left( h\left[ k \right], h^{'}\left[ k \right] \right),$$

where $I\left( a, b \right)=0$ if $a=b$ and $I\left( a,b \right)=1$ otherwise. As the Hamming distance is length-dependent, we define the following $A(h, h^{'})$ as a length-independent distance between haplotype $h$ and $h^{'}$:

$A\left( h, h^{'} \right)=\frac{s(h, h^{'})}{m}$.

Next, let $a=\{h_{1}, h_{2}\}$ and $a^{'}=\{{h^{'}}_{1}, {h^{'}}_{2}\}$ be haplotype pairs to be compared, where $h_{1}, h_{2}, {h^{'}}_{1}, {h^{'}}_{2}\in S^{m}$. We defined the distance between haplotype pairs $a$ and $a^{'}$ as

$$H\left( a, a^{'} \right)=\min\left\{ \frac{A\left( h_{1}, {h^{'}}_{1} \right)+A\left( h_{2}, {h^{'}}_{2} \right)}{2}, \frac{A\left( h_{1}, {h^{'}}_{2} \right)+A\left( h_{2}, {h^{'}}_{1} \right)}{2} \right\}.$$

For unphased-diplotypes $g, g^{'}\in D^{m}$, let $c_{i}=\left\{ h_{i1}, h_{i2} \right\} (1\leq i\leq M)$ and ${c^{'}}_{j}=\left\{ {h^{'}}_{j1}, {h^{'}}_{j2} \right\} (1\leq j\leq M^{'})$ be the *i*-th and the *j*-th candidate haplotype-diplotypes for $g$ and $g^{'}$ respectively. $M$ and $M^{'}$ are the numbers of haplotype-diplotype candidates for $g$ and $g^{'}$ respectively. If we were given a population model $M$, we can compute probability $Prob(c|g,M)$ that haplotype-diplotype candidate $c$ is correct for the haplotype-diplotype data $g$. Let $p_{i}=Prob(c_{i}|g,M)$ and ${p^{'}}_{j}=Prob({c'}_{j}|g,M)$ be the conditional probabilities of the candidate haplotype-diplotype $c_{i}$ and ${c^{'}}_{j}$ under the population model $M$, respectively. We considered hidden Markov model (HMM) defined in HIT algorithm [1] as the population model. Then we defined HIT HMM-based distance (HHD) between two haplotype-diplotypes $g$ and $g^{'}$ is defined as follows:

$$HHD\left( g, g^{'} \right)=\sum_{i=1}^{M} \sum_{j=1}^{M^{'}} H\left( c_{i}, {c^{'}}_{j} \right)\cdot q_{i}\cdot{q^{'}}_{j},$$

where $q_{i}=p_{i}/(\sum_{k=1}^{M} p_{k})$ and ${q^{'}}_{j}={p^{'}}_{j}/(\sum_{k=1}^{M^{'}} {p^{'}}_{k})$ representing the normalized predicted frequencies of the candidate haplotype-diplotypes $c_{i}$ and ${c^{'}}_{j}$, respectively.

[1] Rastas P, Koivisto M, Mannila H, Ukkonen E. A Hidden Markov Technique for Haplotype Reconstruction. Lect. Notes. Bioinform. 3692. 2005;140-151. doi: 10.1007/11557067_12.
