# Supplementary figures and images for: Revealing phenotype-associated functional differences by genome-wide scan of ancient haplotype blocks
Source: PLoS One. 2017 Apr 26;12(4):e0176530. doi: 10.1371/journal.pone.0176530 (PMC5406033; doi:10.1371/journal.pone.0176530)

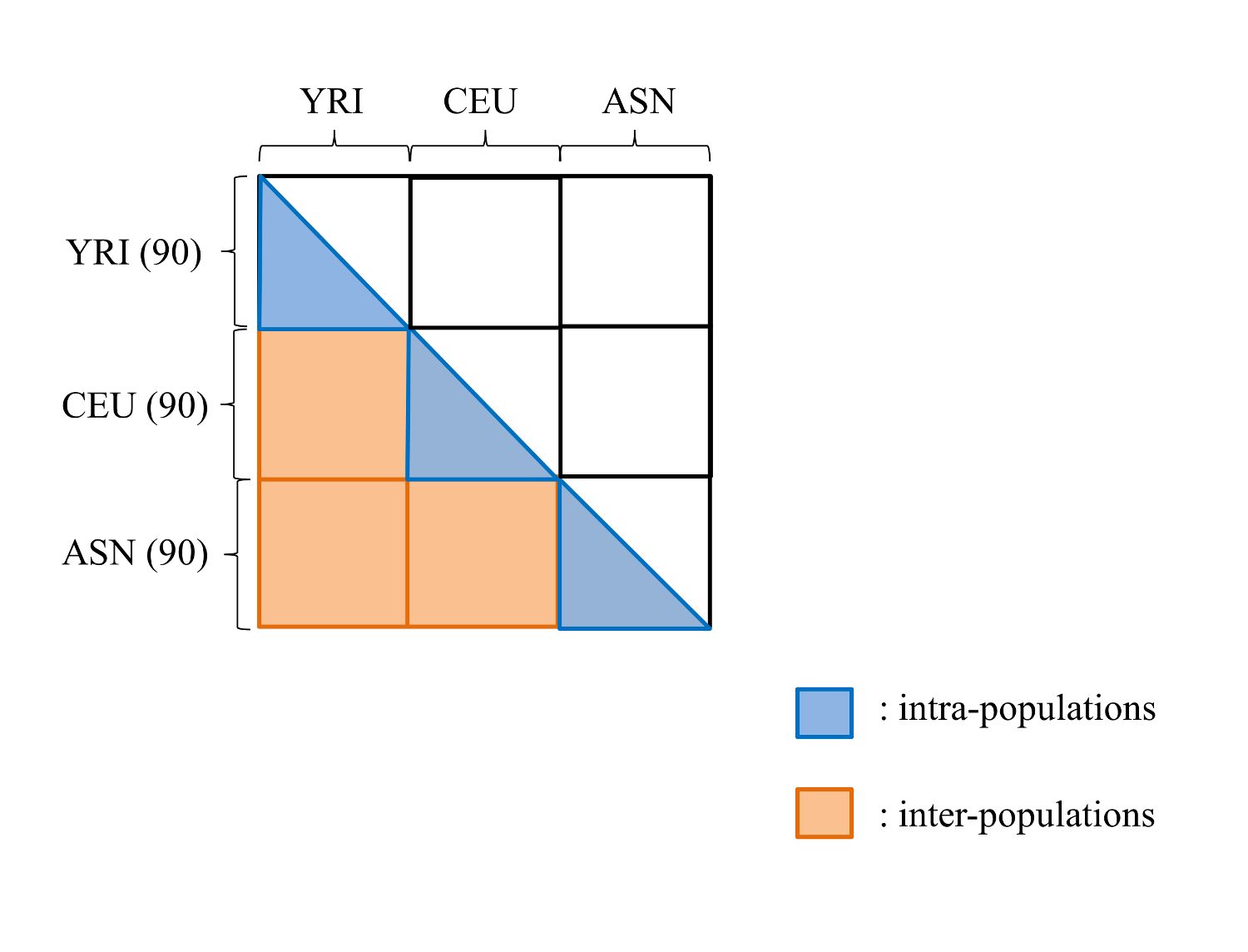

Supplement: S1 Fig — The inter- and intra-population distances in the HHD matrices used to calculate t-statistic scores. (TIF) [file pone.0176530.s002.tif]
