# Supplementary material for: Revealing phenotype-associated functional differences by genome-wide scan of ancient haplotype blocks
Source: PLoS One. 2017 Apr 26;12(4):e0176530. doi: 10.1371/journal.pone.0176530 (PMC5406033; doi:10.1371/journal.pone.0176530)
Supplement: S1 Table — The regions are listed by the t-statistic score assigned by our pipeline. Only the regions that contained genes are shown. (DOCX) [file pone.0176530.s003.docx]

| Score | Chr. | Start position | End position | Genes |
| --- | --- | --- | --- | --- |
| 86.4 | 9 | 123944551 | 123953921 | NDUFA8 |
| 83.28 | 15 | 41410208 | 41604696 | ADAL,ZSCAN29,TUBGCP4,TP53BP1,MAP1A |
| 80.32 | 15 | 32026096 | 32055908 | AVEN |
| 79.75 | 5 | 112189368 | 112219971 | APC |
| 79.44 | 18 | 19655922 | 19713486 | LAMA3 |
| 74.55 | 1 | 65069828 | 65083850 | JAK1 |
| 74.08 | 5 | 1850735 | 1879259 | NDUFS6 |
| 73.78 | 6 | 102245541 | 102268491 | GRIK2 |
| 73.31 | 19 | 38242901 | 38294696 | RHPN2,GPATCH1 |
| 73.06 | 7 | 79671526 | 79691383 | GNAI1 |
| 72.84 | 3 | 121019507 | 121275299 | GSK3B |
| 72.28 | 3 | 50417804 | 50499562 | CACNA2D2 |
| 72.01 | 15 | 47985162 | 48007314 | ATP8B4 |
| 71.82 | 9 | 15933931 | 15992275 | C9orf93 |
| 71.13 | 10 | 25656389 | 25672837 | GPR158 |
| 71.09 | 14 | 63645327 | 63688587 | SYNE2 |
| 70.38 | 16 | 68979732 | 69181984 | ST3GAL2,FUK,COG4,SF3B3 |
| 69.4 | 8 | 25190279 | 25215802 | DOCK5 |
| 69.24 | 5 | 131772473 | 131785379 | LOC441108 |
| 68.86 | 6 | 143632155 | 143637580 | AIG1 |
| 68.83 | 3 | 52829953 | 52871771 | ITIH4,TMEM110 |
| 68.5 | 11 | 26680597 | 26699178 | SLC5A12 |
| 68.23 | 1 | 231831166 | 231833646 | KCNK1 |
| 68.01 | 7 | 2106516 | 2127625 | MAD1L1 |
| 67.56 | 12 | 6337707 | 6338466 | SCNN1A |
| 66.8 | 6 | 116671081 | 116706358 | NT5DC1 |
| 66.78 | 6 | 97690663 | 97750368 | KLHL32,C6orf167 |
| 66.7 | 5 | 145800605 | 145851267 | TCERG1 |
| 66.53 | 5 | 460230 | 465694 | AHRR |
| 66.29 | 20 | 47958234 | 48006194 | SPATA2,RNF114 |
| 65.96 | 5 | 118834319 | 118840649 | HSD17B4 |
| 65.52 | 14 | 56728943 | 56819453 | MUDENG |
| 65.03 | 15 | 29636202 | 29704566 | OTUD7A |
| 64.89 | 1 | 205011575 | 205013520 | IL10 |
| 64.82 | 14 | 69950782 | 70000315 | SYNJ2BP |
| 64.75 | 15 | 78518870 | 78521229 | ARNT2 |
| 64.43 | 4 | 155435821 | 155464121 | DCHS2 |
| 64.39 | 22 | 45649881 | 45665319 | TBC1D22A |
| 64.26 | 5 | 112238314 | 112262448 | REEP5 |
| 64.23 | 1 | 218752203 | 218861725 | MARK1 |
| 64.14 | 17 | 9713768 | 9736460 | GLP2R |
| 63.92 | 1 | 168672987 | 168819281 | GORAB |
| 63.8 | 7 | 7538454 | 7571438 | COL28A1 |
| 63.77 | 10 | 14093666 | 14098918 | FRMD4A |
| 63.7 | 22 | 22501924 | 22509132 | SMARCB1 |
| 63.51 | 15 | 80230095 | 80293845 | EFTUD1 |
| 63.41 | 19 | 40405882 | 40412616 | FAM187B |
| 63.37 | 4 | 106671248 | 106698641 | FLJ20184 |
| 63.37 | 17 | 64552655 | 64568764 | ABCA9 |
| 63.33 | 8 | 14561614 | 14600425 | SGCZ |
| 63.26 | 14 | 35847635 | 35901442 | MBIP |
| 63.24 | 20 | 19232480 | 19286182 | SLC24A3 |
| 62.95 | 11 | 15999048 | 16133464 | SOX6 |
| 62.95 | 21 | 30185479 | 30215280 | GRIK1 |
| 62.88 | 9 | 70730434 | 70736264 | PIP5K1B |
| 62.79 | 2 | 47143532 | 47148792 | TTC7A |
| 62.77 | 10 | 34983712 | 35012850 | PARD3 |
| 62.71 | 20 | 19286645 | 19299929 | SLC24A3 |
| 62.63 | 6 | 167064009 | 167067731 | RPS6KA2 |
| 62.51 | 8 | 26692502 | 26726712 | ADRA1A |
| 62.46 | 4 | 94471706 | 94570269 | GRID2 |
| 62.45 | 3 | 141764774 | 141768194 | CLSTN2 |
| 62.36 | 12 | 110341870 | 110506735 | SH2B3,ATXN2 |
| 62.35 | 14 | 80341951 | 80433377 | C14orf145 |
| 62.15 | 15 | 71136686 | 71223975 | NEO1 |
| 62.13 | 9 | 96083443 | 96096004 | ZNF169 |
| 62.08 | 3 | 124822524 | 124875823 | MYLK |
| 61.99 | 13 | 90931942 | 90933438 | GPC5 |
| 61.93 | 3 | 121596818 | 121611630 | FSTL1 |
| 61.76 | 1 | 11456640 | 11484524 | PTCHD2 |
| 61.68 | 11 | 48071665 | 48114445 | PTPRJ |
| 61.38 | 19 | 8418940 | 8458273 | HNRNPM |
| 61.17 | 11 | 34925688 | 34988152 | PDHX |
| 61.08 | 7 | 94459605 | 94571457 | PPP1R9A |
| 61.06 | 12 | 100400555 | 100417446 | SPIC |
| 61.03 | 7 | 140014377 | 140260681 | BRAF |
| 60.91 | 7 | 127714910 | 127753862 | RBM28 |
| 60.79 | 20 | 9449645 | 9492722 | C20orf103,PAK7 |
| 60.71 | 3 | 30793000 | 30806248 | GADL1 |
| 60.7 | 1 | 203336801 | 203424382 | RBBP5,DSTYK |
| 60.43 | 9 | 15911782 | 15927653 | C9orf93 |
| 60.41 | 1 | 159279214 | 159303096 | USF1,ARHGAP30 |
| 60.25 | 15 | 64047948 | 64053170 | MEGF11 |
| 60.23 | 17 | 25156608 | 25318074 | SSH2 |
| 60.14 | 11 | 83019720 | 83033768 | DLG2 |
| 60.05 | 11 | 62930260 | 62970259 | SLC22A9 |
| 59.96 | 15 | 76716427 | 76733688 | CHRNB4 |
| 59.9 | 1 | 203964468 | 204028762 | NUCKS1,SLC41A1 |
| 59.77 | 20 | 41214300 | 41222873 | PTPRT |
| 59.75 | 18 | 48922319 | 48948096 | DCC |
| 59.66 | 11 | 16136851 | 16177326 | SOX6 |
| 59.64 | 1 | 244312399 | 244340166 | SMYD3 |
| 59.55 | 1 | 75880476 | 76009893 | ACADM |
| 59.48 | 13 | 66276153 | 66328988 | PCDH9 |
| 59.4 | 14 | 60830345 | 60869673 | PRKCH |
| 59.35 | 22 | 41523585 | 41641840 | ARFGAP3,PACSIN2 |
| 59.35 | 14 | 68442503 | 68462017 | ACTN1 |
| 59.25 | 4 | 72332316 | 72342980 | SLC4A4 |
| 59.22 | 11 | 12176480 | 12184257 | MICAL2 |
| 59.21 | 11 | 20663510 | 20687919 | NELL1 |
| 59.2 | 18 | 48961290 | 49003933 | DCC |
| 59.19 | 1 | 64312721 | 64326387 | ROR1 |
| 58.99 | 21 | 25947970 | 25962999 | JAM2 |
| 58.93 | 11 | 83432571 | 83446491 | DLG2 |
| 58.93 | 6 | 38651355 | 38740681 | BTBD9 |
| 58.92 | 6 | 152328398 | 152340128 | ESR1 |
| 58.85 | 2 | 42326398 | 42356444 | EML4 |
| 58.81 | 1 | 116084319 | 116101491 | CASQ2 |
| 58.79 | 12 | 10122660 | 10142287 | CLEC1A |
| 58.77 | 11 | 30427273 | 30444885 | MPPED2 |
| 58.76 | 12 | 79791042 | 79812822 | LIN7A |
| 58.66 | 16 | 56344212 | 56392233 | KATNB1,KIFC3 |
| 58.48 | 12 | 130977857 | 131110596 | EP400 |
| 58.44 | 14 | 63884064 | 63940963 | MTHFD1 |
| 58.37 | 3 | 85888887 | 85932752 | CADM2 |
| 58.36 | 6 | 170459223 | 170529596 | FAM120B |
| 58.35 | 22 | 42372045 | 42383885 | EFCAB6 |
