# Supplementary material for: Revealing phenotype-associated functional differences by genome-wide scan of ancient haplotype blocks
Source: PLoS One. 2017 Apr 26;12(4):e0176530. doi: 10.1371/journal.pone.0176530 (PMC5406033; doi:10.1371/journal.pone.0176530)
Supplement: S2 Table — The genes are listed by cluster. (DOCX) [file pone.0176530.s004.docx]

| Cluster | Genes |
| --- | --- |
| 2 | ADAL, ARHGAP30, BTBD9, CLEC1A, DOCK5, ESR1, FAM187B, GADL1, GLP2R, GORAB, GPATCH1, GPR158, GSK3B, HSD17B4, LAMA3, MAP1A, MBIP, MICAL2, MPPED2, MYLK, NDUFS6, NUCKS1, OTUD7A, PTCHD2, PTPRT, RHPN2, SCNN1A, SLC41A1, SLC4A4, SYNJ2BP, TP53BP1, TUBGCP4, USF1, ZSCAN29 |
| 3 | ACTN1, ADRA1A, ARFGAP3, ATXN2, CHRNB4, CLSTN2, FSTL1, IL10, JAM2, MEGF11, PACSIN2, SH2B3, SLC22A9, SOX6, SPIC, TCERG1, TTC7A |
| 4 | ACADM, ATP8B4, C6orf167, EML4, FAM120B, KLHL32, NELL1, PPP1R9A, SMYD3 |
| 5 | ABCA9, AHRR, AIG1, APC, ARNT2, AVEN, BRAF, C14orf145, C20orf103, C9orf93, CACNA2D2, CADM2, CASQ2, COG4, COL28A1, DCC, DCHS2, DLG2, DSTYK, EFCAB6, EFTUD1, EP400, FLJ20184, FRMD4A, FUK, GNAI1, GPC5, GRID2, GRIK1, GRIK2, HNRNPM, ITIH4, JAK1, KATNB1, KCNK1, KIFC3, LIN7A, LOC441108, MAD1L1, MARK1, MTHFD1, MUDENG, NDUFA8, NEO1, NT5DC1, PAK7, PARD3, PCDH9, PDHX, PIP5K1B, PRKCH, PTPRJ, RBBP5, RBM28, REEP5, RNF114, ROR1, RPS6KA2, SF3B3, SGCZ, SLC24A3, SLC5A12, SMARCB1, SPATA2, SSH2, ST3GAL2, SYNE2, TBC1D22A, TMEM110, ZNF169 |
