# Supplementary material for: Revealing phenotype-associated functional differences by genome-wide scan of ancient haplotype blocks
Source: PLoS One. 2017 Apr 26;12(4):e0176530. doi: 10.1371/journal.pone.0176530 (PMC5406033; doi:10.1371/journal.pone.0176530)
Supplement: S3 Table — The t-statistic score profiles used for clustering are shown in the second, third, and fourth columns; each was calculated by pair-wise comparisons of populations. The fifth column shows the cluster number assigned by clustering. The sixth column shows the t-statistic score calculated for the three populations. The last column shows the genes in each haplotype block. The ID of each ancient haplotype block is the rs number of the first SNP in the block. Only the top 1% of ancient haplotype blocks that contained genes are shown here. (DOCX) [file pone.0176530.s005.docx]

| ID | CEU-YRI | CEU-ASN | ASN-YRI | Cluster | three pops | Genes |
| --- | --- | --- | --- | --- | --- | --- |
| rs1556217 | 76.63 | 27.53 | 84.68 | 5 | 86.4 | NDUFA8 |
| rs3742971 | 113.74 | 36.36 | 39.19 | 2 | 83.28 | ADAL ZSCAN29 TUBGCP4 TP53BP1 MAP1A |
| rs525243 | 95.82 | 19.75 | 54.5 | 5 | 80.32 | AVEN |
| rs2546108 | 72.73 | 21.97 | 90.95 | 5 | 79.75 | APC |
| rs12957168 | 84.2 | 37.03 | 47.74 | 2 | 79.44 | LAMA3 |
| rs1048007 | 93.99 | 20.1 | 52.95 | 5 | 74.55 | JAK1 |
| rs2276986 | 73.09 | 63.37 | 24.57 | 2 | 74.08 | NDUFS6 |
| rs2786254 | 70.67 | 10.58 | 72.66 | 5 | 73.78 | GRIK2 |
| rs1559088 | 95.99 | 19.91 | 43.59 | 2 | 73.31 | RHPN2 GPATCH1 |
| rs4731273 | 61.46 | 12.54 | 81.58 | 5 | 73.06 | GNAI1 |
| rs6438550 | 77.74 | 34.37 | 49.02 | 2 | 72.84 | GSK3B |
| rs2282755 | 66.65 | 30.7 | 54.87 | 5 | 72.28 | CACNA2D2 |
| rs12440932 | 36.97 | 52.31 | 73.41 | 4 | 72.01 | ATP8B4 |
| rs10810488 | 64.37 | 5.46 | 84.15 | 5 | 71.82 | C9orf93 |
| rs16925561 | 70.41 | 54.91 | 21.9 | 2 | 71.13 | GPR158 |
| rs17101669 | 73.52 | 8.71 | 63.05 | 5 | 71.09 | SYNE2 |
| rs4985526 | 84.86 | 10.92 | 58.33 | 5 | 70.38 | ST3GAL2 FUK COG4 SF3B3 |
| rs2666156 | 85.61 | 21.97 | 35.09 | 2 | 69.4 | DOCK5 |
| rs1016988 | 47.69 | 31.36 | 67.79 | 5 | 69.24 | LOC441108 |
| rs7765576 | 51.19 | 34.56 | 60.22 | 5 | 68.86 | AIG1 |
| rs2535646 | 68.7 | 9.46 | 65.34 | 5 | 68.83 | ITIH4 TMEM110 |
| rs12289000 | 97.41 | 6.71 | 62.89 | 5 | 68.5 | SLC5A12 |
| rs12407694 | 89.06 | 15.61 | 63.87 | 5 | 68.23 | KCNK1 |
| rs3778969 | 50.63 | 10.18 | 89.36 | 5 | 68.01 | MAD1L1 |
| rs10849445 | 85.65 | 29.28 | 26.13 | 2 | 67.56 | SCNN1A |
| rs1204826 | 38.19 | 24.77 | 86.7 | 5 | 66.8 | NT5DC1 |
| rs1206144 | 34.09 | 28.22 | 77.19 | 4 | 66.78 | KLHL32 C6orf167 |
| rs6889741 | 46.49 | 70.84 | 23.5 | 3 | 66.7 | TCERG1 |
| rs732111 | 66.8 | 24.74 | 47.43 | 5 | 66.53 | AHRR |
| rs2769982 | 43.17 | 24.94 | 74.02 | 5 | 66.29 | SPATA2 RNF114 |
| rs257973 | 59.93 | 46.77 | 40.43 | 2 | 65.96 | HSD17B4 |
| rs8011432 | 67.55 | 5.3 | 59.15 | 5 | 65.52 | MUDENG |
| rs11630385 | 77.83 | 26.12 | 35.62 | 2 | 65.03 | OTUD7A |
| rs3021094 | 8.34 | 69.05 | 59.41 | 3 | 64.89 | IL10 |
| rs4902815 | 67.79 | 50.29 | 20.32 | 2 | 64.82 | SYNJ2BP |
| rs1037124 | 51.37 | 2.88 | 77.92 | 5 | 64.75 | ARNT2 |
| rs10003606 | 74.21 | 2.64 | 55.34 | 5 | 64.43 | DCHS2 |
| rs131864 | 75.2 | 3.94 | 50.04 | 5 | 64.39 | TBC1D22A |
| rs419155 | 50.38 | 17.66 | 75.61 | 5 | 64.26 | REEP5 |
| rs17007868 | 58.76 | 17.52 | 64.08 | 5 | 64.23 | MARK1 |
| rs9674995 | 59.22 | 47.23 | 36.03 | 2 | 64.14 | GLP2R |
| rs12128140 | 51.31 | 43.89 | 46.39 | 2 | 63.92 | GORAB |
| rs2108065 | 69.91 | 0.56 | 65.31 | 5 | 63.8 | COL28A1 |
| rs12571559 | 44.22 | 30.12 | 60.69 | 5 | 63.77 | FRMD4A |
| rs6003899 | 69.95 | 4.24 | 62.95 | 5 | 63.7 | SMARCB1 |
| rs16973420 | 62.16 | 6.13 | 74.49 | 5 | 63.51 | EFTUD1 |
| rs10416023 | 59.41 | 64.64 | 12.33 | 2 | 63.41 | FAM187B |
| rs3960769 | 43.15 | 23.5 | 71.14 | 5 | 63.37 | FLJ20184 |
| rs10512523 | 53.59 | 19.14 | 60.77 | 5 | 63.37 | ABCA9 |
| rs11203648 | 75.01 | 19.41 | 46.39 | 5 | 63.33 | SGCZ |
| rs17175276 | 58.32 | 45.7 | 35.31 | 2 | 63.26 | MBIP |
| rs6035283 | 63.29 | 14.81 | 59.29 | 5 | 63.24 | SLC24A3 |
| rs16932469 | 32.57 | 60.5 | 43.04 | 3 | 62.95 | SOX6 |
| rs2832478 | 79.03 | 6.41 | 47.28 | 5 | 62.95 | GRIK1 |
| rs10781282 | 66.27 | 21.93 | 44.54 | 5 | 62.88 | PIP5K1B |
| rs4953454 | 25.4 | 66.65 | 36.93 | 3 | 62.79 | TTC7A |
| rs12241700 | 66.87 | 10.92 | 70.43 | 5 | 62.77 | PARD3 |
| rs7264626 | 62.89 | 4.88 | 58.72 | 5 | 62.71 | SLC24A3 |
| rs7773974 | 61.51 | 2.32 | 68.67 | 5 | 62.63 | RPS6KA2 |
| rs4732646 | 37.55 | 66.83 | 25.36 | 3 | 62.51 | ADRA1A |
| rs10002068 | 67.49 | 2.71 | 68.05 | 5 | 62.46 | GRID2 |
| rs4683509 | 35.81 | 72.91 | 21.27 | 3 | 62.45 | CLSTN2 |
| rs2078863 | 24.79 | 77.32 | 32.77 | 3 | 62.36 | SH2B3 ATXN2 |
| rs10141192 | 44.39 | 24.11 | 69.52 | 5 | 62.35 | C14orf145 |
| rs11632639 | 63.77 | 22.04 | 47.9 | 5 | 62.15 | NEO1 |
| rs7874441 | 43.43 | 17.65 | 74.9 | 5 | 62.13 | ZNF169 |
| rs3845915 | 78.79 | 54.77 | 10.89 | 2 | 62.08 | MYLK |
| rs9560814 | 37.09 | 16.1 | 81.71 | 5 | 61.99 | GPC5 |
| rs13709 | 21.34 | 89.78 | 23.78 | 3 | 61.93 | FSTL1 |
| rs10159458 | 65.28 | 27.24 | 34.58 | 2 | 61.76 | PTCHD2 |
| rs3942852 | 61.68 | 0.36 | 69.11 | 5 | 61.68 | PTPRJ |
| rs17160491 | 67.63 | 0.8 | 70.88 | 5 | 61.38 | HNRNPM |
| rs11539202 | 70.2 | 11.69 | 46.91 | 5 | 61.17 | PDHX |
| rs854745 | 30.3 | 46.37 | 57.41 | 4 | 61.08 | PPP1R9A |
| rs11110820 | 8.03 | 61.89 | 59.32 | 3 | 61.06 | SPIC |
| rs7792746 | 64.34 | 7.44 | 60.1 | 5 | 61.03 | BRAF |
| rs1545444 | 36.9 | 9.08 | 82.1 | 5 | 60.91 | RBM28 |
| rs2283635 | 60.36 | 17.82 | 54.88 | 5 | 60.79 | C20orf103 PAK7 |
| rs9882060 | 87.18 | 32.16 | 13.51 | 2 | 60.71 | GADL1 |
| rs11578293 | 35.49 | 20.09 | 77.5 | 5 | 60.7 | RBBP5 DSTYK |
| rs1536685 | 47.28 | 6.96 | 81.14 | 5 | 60.43 | C9orf93 |
| rs2073655 | 77.89 | 38.82 | 12.28 | 2 | 60.41 | USF1 ARHGAP30 |
| rs333556 | 38.23 | 74.34 | 17.69 | 3 | 60.25 | MEGF11 |
| rs8081325 | 44.26 | 15.71 | 73.26 | 5 | 60.23 | SSH2 |
| rs6592134 | 37.47 | 16.43 | 77.14 | 5 | 60.14 | DLG2 |
| rs7101446 | 7.58 | 65.63 | 48.53 | 3 | 60.05 | SLC22A9 |
| rs12441998 | 45.24 | 69.64 | 12.35 | 3 | 59.96 | CHRNB4 |
| rs1620334 | 69.55 | 30.48 | 31.47 | 2 | 59.9 | NUCKS1 SLC41A1 |
| rs2000135 | 49.4 | 45.82 | 28.02 | 2 | 59.77 | PTPRT |
| rs9956477 | 41.38 | 12.77 | 76.11 | 5 | 59.75 | DCC |
| rs16932650 | 24.78 | 60.87 | 40.82 | 3 | 59.66 | SOX6 |
| rs2788019 | 32.57 | 40.34 | 57.11 | 4 | 59.64 | SMYD3 |
| rs12732722 | 31.51 | 34.19 | 66.17 | 4 | 59.55 | ACADM |
| rs9317615 | 67.98 | 2.28 | 61.3 | 5 | 59.48 | PCDH9 |
| rs7158527 | 74.08 | 1.85 | 56 | 5 | 59.4 | PRKCH |
| rs4393836 | 29.48 | 69.3 | 23.71 | 3 | 59.35 | ARFGAP3 PACSIN2 |
| rs4899269 | 12.35 | 82.1 | 30.02 | 3 | 59.35 | ACTN1 |
| rs2045012 | 73.65 | 44.31 | 10.42 | 2 | 59.25 | SLC4A4 |
| rs17477991 | 71.92 | 48.97 | 6.75 | 2 | 59.22 | MICAL2 |
| rs1793000 | 19.43 | 41.14 | 64.28 | 4 | 59.21 | NELL1 |
| rs8083850 | 38.6 | 23.44 | 68.53 | 5 | 59.2 | DCC |
| rs1772618 | 29.5 | 14.78 | 93.22 | 5 | 59.19 | ROR1 |
| rs9974110 | 38.12 | 65.83 | 17.98 | 3 | 58.99 | JAM2 |
| rs1384751 | 64.07 | 1.17 | 59.8 | 5 | 58.93 | DLG2 |
| rs17543178 | 45.42 | 42.22 | 40.79 | 2 | 58.93 | BTBD9 |
| rs985695 | 55.1 | 36.32 | 37.11 | 2 | 58.92 | ESR1 |
| rs17671409 | 29 | 23.1 | 75.28 | 4 | 58.85 | EML4 |
| rs7544242 | 70.7 | 18.18 | 40.73 | 5 | 58.81 | CASQ2 |
| rs11053573 | 50.87 | 44.86 | 29.8 | 2 | 58.79 | CLEC1A |
| rs11031094 | 66.32 | 19.34 | 36.72 | 2 | 58.77 | MPPED2 |
| rs1716546 | 79.83 | 6.89 | 41.27 | 5 | 58.76 | LIN7A |
| rs2965798 | 42.84 | 36.26 | 46.74 | 5 | 58.66 | KATNB1 KIFC3 |
| rs7978708 | 61.36 | 8.19 | 53.46 | 5 | 58.48 | EP400 |
| rs1256112 | 44.05 | 16.12 | 68.97 | 5 | 58.44 | MTHFD1 |
| rs12714637 | 48.45 | 23.71 | 64.32 | 5 | 58.37 | CADM2 |
| rs9356637 | 18.43 | 53.84 | 59.19 | 4 | 58.36 | FAM120B |
| rs137744 | 29.77 | 15.82 | 75.38 | 5 | 58.35 | EFCAB6 |
