# Supplementary material for: Revealing phenotype-associated functional differences by genome-wide scan of ancient haplotype blocks
Source: PLoS One. 2017 Apr 26;12(4):e0176530. doi: 10.1371/journal.pone.0176530 (PMC5406033; doi:10.1371/journal.pone.0176530)
Supplement: S4 Table — Genes that were previously reported to be affected by positive selection in the top 1% of ancient haplotype blocks are shown for each cluster. The genes are also classified according to the population where the positive selection was detected. (DOCX) [file pone.0176530.s006.docx]

| Cluster | #genes | #previously reported genes | Reported genes | | |
| --- | --- | --- | --- | --- | --- |
|  |  |  | YRI | CEU | ASN |
| 2 | 34 | 9 | ARHGAP30  MYLK  USF1 | ADAL  MAP1A  MYLK  SYNJ2BP  TP53BP1  TUBGCP4  ZSCAN29 | ADAL  MAP1A  TP53BP1  TUBGCP4  ZSCAN29 |
| 3 | 17 | 8 | ACTN1 | CLSTN2  SPIC  TCERG1 | ACTN1  ATXN2  FSTL1  MEGF11  SH2B3 |
| 4 | 9 | 2 | - | - | ACADM  EML4 |
| 5 | 70 | 14 | EFTUD1  PRKCH | APC  DCC  DLG2  FLJ20184  NDUFA8  PDHK  PRKCH  SLC24A3 | APC  DCC  DLG2  FLJ20184  GPC5  GRID2  REEP5  ROR1  TBC1D22A |
