# Supplementary material for: Revealing phenotype-associated functional differences by genome-wide scan of ancient haplotype blocks
Source: PLoS One. 2017 Apr 26;12(4):e0176530. doi: 10.1371/journal.pone.0176530 (PMC5406033; doi:10.1371/journal.pone.0176530)
Supplement: S5 Table — SNPs in the top 1% of the ancient haplotype blocks that were previously reported [55–72] to be associated with phenotypes by GWAS are listed for each cluster. Each SNP is reported with the genes that the SNP is located in or linked with. The SNPs are mapped to the biological pathways through the reported genes. In addition to the reported genes, genes mapped to the biological pathways in the top 1% of ancient haplotype blocks were also listed in the sixth column. (DOCX) [file pone.0176530.s007.docx]

| Cluster | SNP in our  result | Reported gene(s) | Trait/Disease | Ancestry | KEGG pathway map | Mapped genes |
| --- | --- | --- | --- | --- | --- | --- |
| 2 | rs10416218 | GPATCH1 | Bone mineral density | European  East Asian | - | - |
|  | rs823123 | NUCKS1, RAB7L1 | Prostate-specific antigen levels | Korean | - | - |
|  | rs6679073 | SLC41A1 | Prostate-specific antigen levels | Korean | - | - |
|  | rs7586898 | AC113608.1 | Hair morphology | European | - | - |
|  | rs9383951 | ESR1 | Breast cancer | Chinese | 04915 Estrogen signaling pathway  04917 Prolactin signaling pathway  04919 Thyroid hormone signaling pathway  04961 Endocrine and other factor-regulated calcium reabsorption  05205 Proteoglycans in cancer | ESR1, GNAI1  ESR1, GSK3B  ESR1, GSK3B  ESR1  BRAF, ESR1 |
| 3 | rs11949289 | intergenic | Response to anti-depressant treatment in major depressive disorder | European  African American | - | - |
|  | rs7101446 | SLC22A9 | Economic and political preferences | European | - | - |
| 4 | rs4949874 | ACADM | Blood metabolite ratios | European | 00640 Propanoate metabolism  00071 Fatty acid degradation  00280 Valine, leucine and isoleucine degradation  00410 beta-Alanine metabolism  03320 PPAR signaling pathway | ACADM  ACADM  ACADM  ACADM, GADL1  ACADM |
| 5 | rs3818638 | NDUFA8 | Obesity-related traits | Hispanic | 00190 Oxidative phosphorylation  05010 Alzheimer's disease  05012 Parkinson's disease  05016 Huntington's disease  04932 Non-alcoholic fatty liver disease (NAFLD) | NDUFA8, NDUFS6  GSK3B, NDUFS8, NDUFS6  GNAI1, NDUFA8, NDUFS6  NDUA8, NDUFS6  GSK3B, NDUFA8, NDUFS6 |
|  | rs6475606 | CDKN2B-AS1 | Intracranial aneurysm | European | 04350 TGF-beta signaling pathway  04068 FoxO signaling pathway  04110 Cell cycle  05203 Viral carcinogenesis  05222 Small cell lung cancer  05166 HTLV-I infection | BRAF, IL10  ACTN1, JAK1, MAD1L1  LAMA3  APC, GSK3B, JAK1 |
|  | rs10965235 | CDKN2BAS | Endometriosis | Japanese | - | - |
|  | rs1016988 | IRF1, SLC22A4, SLC22A5 | Fibrinogen | European | (IRF1)  04917 Prolactin signaling pathway  05133 Pertussis  05160 Hepatitis C  (SLC22A4, SLC22A5)  05231 Choline metabolism in cancer | ESR1, GSK3B  GNAI1, IL10  BRAF, GSK3B, JAK1  PIP5K1B |
|  | rs11242111 | IRF1, C5orf56 | Fibrinogen | European | - | - |
|  | rs11118346 | LYPLAL1 | Height | European | - | - |
|  | rs2820446 | LYPLAL1 | Type 2 diabetes | European  East Asian  South Asian  Mexican | - | - |
|  | rs10056340 | CAMK4, TSLP, WDR36, SLC25A46 | Allergic sensitization | European | (CAMK4)  04020 Calcium signaling pathway  04024 cAMP signaling pathway  04921 Oxytocin signaling pathway  04725 Cholinergic synapse  04720 Long-term potentiation  04722 Neurotrophin signaling pathway  04380 Osteoclast differentiation  05031 Amphetamine addiction  05034 Alcoholism  (TSLP)  04630 Jak-STAT signaling pathway  04060 Cytokine-cytokine receptor interaction  (WDR36)  03008 Ribosome biogenesis in eukaryotes | ADRA1A, MYLK  BRAF, GNAI1  CACNA2D2, GNAI1, MYLK  CHRNB4, GNAI1  BRAF, RPS6KA2  BRAF, GSK3B, RPS6KA2, SH2B3  JAK1  BRAF, GNAI1  IL10, JAK1  IL10  EFTUD1, RBM28 |
|  | rs7620363 | NR | Non-substance related behavioral disinhibition | European | - |  |
|  | rs3008706 | intergenic | Bilirubin levels | Korean | - | - |
|  | rs11203649 | SGCZ | Obesity-related traits | Hispanic | - | - |
|  | rs3942852 | PTPRJ | Acute lymphoblastic leukemia (childhood) | European | 04520 Adherens junction | ACTN1, PARD3, PTPRJ |
