# Supplementary material for: Revealing phenotype-associated functional differences by genome-wide scan of ancient haplotype blocks
Source: PLoS One. 2017 Apr 26;12(4):e0176530. doi: 10.1371/journal.pone.0176530 (PMC5406033; doi:10.1371/journal.pone.0176530)
Supplement: S6 Table — There were no immune system-related genes in Cluster 4. However, there were some genes related to metabolism. (DOCX) [file pone.0176530.s008.docx]

| Category |  | Pathway | Mapped gene |
| --- | --- | --- | --- |
| Metabolism | Amino acid metabolism | Valine, leucine and isoleucine degradation | ACADM |
|  | Carbohydrate metabolism | Propanoate metabolism | ACADM |
|  | Lipid metabolism | Fatty acid degradation | ACADM |
|  | Metabolism of other amino acids | beta-Alanine metabolism | ACADM |
| Organismal Systems | Endocrine system | PPAR signaling pathway | ACADM |
| Human Diseases | Cancers | Non-small cell lung cancer | EML4 |
